# All-cause and cause-specific mortality in respiratory symptom clusters: a population-based multicohort study

**DOI:** 10.1186/s12931-025-03224-7

**Published:** 2025-04-16

**Authors:** Daniil Lisik, Helena Backman, Hannu Kankaanranta, Rani Basna, Linnea Hedman, Linda Ekerljung, Fredrik Nyberg, Anne Lindberg, Göran Wennergren, Eva Rönmark, Bright Nwaru, Lowie Vanfleteren

**Affiliations:** 1 Krefting Research Centre, Institute of Medicine, Sahlgrenska Academy, University of Gothenburg, Gothenburg, Sweden; 2 Department of Public Health and Clinical Medicine, Section of Sustainable Health, The OLIN Unit, Umeå University, Umeå, Sweden; 3 Tampere University Respiratory Research Group, Faculty of Medicine and Health Technology, Tampere University, Tampere, Finland; 4 Department of Respiratory Medicine, Seinäjoki Central Hospital, Seinäjoki, Finland; 5 Division of Geriatric Medicine, Department of Clinical Sciences in Malmö, Lund University, Malmö, Sweden; 6 School of Public Health and Community Medicine, Institute of Medicine, Sahlgrenska Academy, University of Gothenburg, Gothenburg, Sweden; 7 Department of Public Health and Clinical Medicine, The OLIN Unit, Umeå University, Umeå, Sweden; 8 Department of Pediatrics, Sahlgrenska Academy, University of Gothenburg, Gothenburg, Sweden; 9 Wallenberg Centre for Molecular and Translational Medicine, University of Gothenburg, Gothenburg, Sweden; 10 COPD Center, Department of Respiratory Medicine and Allergology, Sahlgrenska University Hospital, Gothenburg, Sweden; 11 Department of Internal Medicine and Clinical Nutrition, Institute of Medicine, Sahlgrenska Academy, University of Gothenburg, Gothenburg, Sweden

**Keywords:** Cluster analysis, Cough, Dyspnea, Machine learning, Mortality, Respiratory symptoms, Wheezing

## Abstract

**Background:**

Respiratory symptoms are common in the general adult population. Increased burden of respiratory symptoms may increase the risk of mortality. We assessed the association between respiratory symptom clusters and mortality.

**Methods:**

Participants were derived from two population-based Swedish adult cohorts (*N* = 63,060). Cluster analysis was performed with Locality Sensitive Hashing (LSH)-*k-*prototypes in subjects with ≥ 1 self-reported respiratory symptom. Linked mortality register data (up to 21 years of follow-up, > 600,000 person-years) were used. Associations between clusters and all-cause/cause-specific mortality were assessed using asymptomatic subjects as reference.

**Results:**

Over 60% reported ≥ 1 respiratory symptom and ~ 30% reported ≥ 5 respiratory symptoms. Five clusters were identified, partly overlapping with established respiratory disease phenotypes but many individuals were undiagnosed: (1) "low-symptomatic" (30.3%); (2) "allergic nasal symptoms" (10.7%); (3) "allergic nasal symptoms, wheezing, and dyspnea attacks" (4.7%); (4) "wheezing and dyspnea attacks" (6.6%); (5) "recurrent productive cough and wheezing" (4.1%). All but Cluster 2 were associated with all-cause mortality, highest risk for Cluster 3 (hazard ratio 1.4, 95% confidence interval 1.13–1.73) and Cluster 5 (1.4, 1.22–1.61). Comparable associations were seen for cardiovascular mortality. For respiratory mortality, Cluster 4 (2.02, 1.18–3.46) and Cluster 5 (1.89, 1.1–3.25) were most strongly associated.

**Conclusions:**

Respiratory symptoms are common in the general adult population, with identifiable clusters. These clusters have clinical relevancy as they are differentially associated with mortality and relatively weakly correlated with diagnosed respiratory disease.

**Supplementary Information:**

The online version contains supplementary material available at 10.1186/s12931-025-03224-7.

## Introduction

Roughly half of adults in Europe report respiratory symptoms, such as cough, shortness of breath, and wheezing [1, 2]. However, many of these do not have a diagnosis of respiratory disease. Partly, this is due to underdiagnosis [3], but given the heterogeneity in clinical presentation of obstructive lung diseases, there may also be phenotypes that are yet to be clearly defined. It is of great importance to elucidate patterns of respiratory symptoms given their negative health impact. Respiratory symptoms have been linked to impaired lung function and accelerated loss thereof [4], as well as cardiovascular comorbidity [5]. Recent reports also indicate that respiratory symptoms, such as attacks of breathlessness and wheezing, are associated with higher risk of mortality [6]. Detailed characterization of respiratory symptoms is of importance; for example, productive but not dry chronic cough has been associated with increased mortality [7]. It is thus likely that specific patterns of respiratory symptoms are also differentially associated with mortality.

Machine learning (ML)-based phenotyping is increasingly utilized in research, particularly with so-called unsupervised cluster analysis, as it enables discovery of latent patterns in high-dimensional data, with theoretically less bias than the historically common practice of deriving disease subtypes through clinical experience [8, 9]. Previous ML-based work has identified meaningful and distinct phenotypes of e.g., asthma [10] and chronic obstructive pulmonary disease (COPD) [11]. However, such analyses have been limited to subjects with pre-defined and diagnosed disease. The aim of the present study was to identify and characterize clusters using ML based exclusively on the presence/absence of respiratory symptoms in a representative adult population. Furthermore, we assessed associations between the derived clusters and mortality to determine their clinical significance.

## Methods

### Study design and participants

**Fig. 1:**
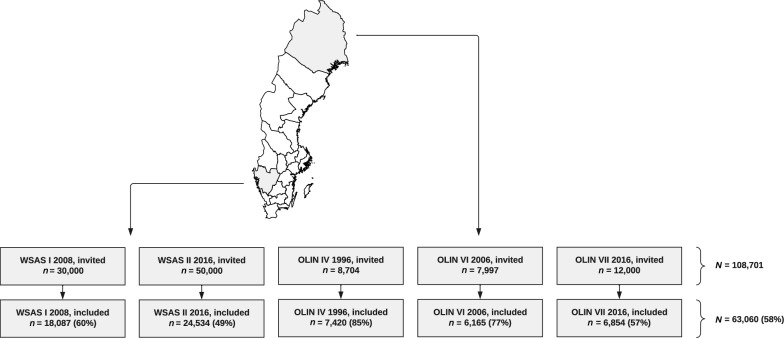
Regions and participation rates of included cohorts. The Obstructive Lung Disease in Northern Sweden (OLIN) studies were performed in Norrbotten county, Sweden (green boxes/shaded area in map). The West Sweden Asthma Study (WSAS) was performed in Västra Götaland county, Sweden (blue boxes/shaded area in map). Percentages denote the proportion of invited individuals

Two Swedish cohorts—West Sweden Asthma Study (WSAS; *N* = 42,621) from the southwestern county of Västra Götaland, and the Obstructive Lung Disease in Northern Sweden (OLIN; *N* = 20,439) from the northernmost county of Norrbotten—were used (Fig. 1). WSAS and OLIN consist of multiple recruitment-wave population-based cohorts, in which adults living in the study areas were randomly selected. Enrolment age was 16–75 years in WSAS and 20–74 in OLIN. Participating subjects completed postal surveys based on validated questionnaires, extensively used in previous publications [12, 13]. The study was approved by the Ethics Committee at the University of Gothenburg (034-08 and 052-16) for WSAS and the Ethics Committee at Umeå University (1996-123, 2005-157M, 2015-404-31, and 2017-393-32M) for OLIN. Informed written consent was obtained from all participants. The writing of this manuscript was done in alignment with the Strengthening the Reporting of Observational Studies in Epidemiology (STROBE) guideline (Supplementary document).

### Data sources and variable definitions

To maximize the data-driven aspect of the cluster analysis, we included a wide range of respiratory symptoms, covering five domains: wheezing, dyspnea, coughing, night-time respiratory symptoms, and nasal/sinus symptoms. These data were extracted from the postal surveys. We included only questions that did not necessitate diagnosis of any disease for an affirmative response. DL, HB, HK, BN, and LV selected the variables to include in an informal process, aiming to maximize the comprehensiveness of clinical characterization, while excluding clinically/conceptually overlapping variables and variables for which there is no apparent relevance (https://osf.io/jq3ns). The questions were binary, inquiring about the presence of various symptoms, typically within the past 12 months (https://osf.io/h48es). Background characteristics were also obtained from the surveys, covering family history of asthma/COPD/allergy, sociodemographic variables, and environmental exposures (https://osf.io/jsf4k). While the surveys were similar, some cohort surveys lacked certain questions, particularly on rhinitis symptoms within OLIN. Overall missingness, however, was 11.2% (Supplementary Fig. 1). Several respiratory symptom variables were correlated and/or inquired about clinically overlapping aspects of respiratory health (Supplementary Fig. 2). For this reason, a feature selection was performed based on domain expertise by DL and LV to eliminate redundant variables. Furthermore, as some variables demonstrated a clear ordinal pattern of e.g., increasing chronicity or symptom severity, selected variables were combined to ordinal composite variables (https://osf.io/hykfz and https://osf.io/jq3ns). Comorbidity variables were extracted from the National Patient Register data (comprehensively covering specialist outpatient/in-patient healthcare), and were used for further characterization and for confounder adjustment in the mortality analyses. In light of limited sample size for the mortality analyses, particularly the subgroup analyses and cause-specific analyses, an overall measure of comorbidity burden was calculated using the Charlson Comorbidity index (CCI) [14], adapted for the Swedish register-based research context [15] and further modified by explicitly adjusting for the major respiratory comorbidities of asthma and COPD. CCI was calculated based on the International Classification of Diseases, version 10 (ICD-10) codes recorded for any specialist outpatient/in-patient healthcare contact during the year of the survey or (up to 11 years) earlier. Data on mortality were derived from the National Cause-of-Death Register [16], which comprehensively covers deaths in the Swedish population, including the underlying cause(s) of death through ICD-10 codes. Subjects without a record were considered to have survived until the end of the follow-up. In addition to all-cause mortality, we investigated cardiovascular, respiratory, and lung cancer mortality specifically as outcomes (Supplementary Table 1), as these are major causes of death in the general adult population and have previously been associated with respiratory symptoms [17, 18].

### Statistical analysis

Register data utilized in the present study have near-complete coverage, thus requiring no imputation. Missing data in the postal surveys, however, were imputed using multiple imputation by chained equations with random forests, to enable use of all subjects/cohorts. One hundred datasets were generated. The imputed data were pooled through the mode for categorical variables and the mean for continuous variables for descriptive statistics (Supplementary text 1).

Cluster analysis was performed using a modified implementation of Locality-Sensitive Hashing (LSH)-*k*-representatives (LSH-*k*-prototypes), to accommodate the mixed binary and ordinal variables. This algorithm is suitable for high-dimension categorical data, and additionally addresses accuracy/stability issues of previous related algorithms such as *k*-modes [19]. For each imputed dataset, the clustering algorithm was run in subjects with ≥ 1 respiratory symptom, assessing solutions of 2–10 clusters. Based on the statistical metrics and clinical evaluation, five clusters were deemed to be optimal. Details are provided in Supplementary text 2. For descriptive statistics, the cluster labels in the imputed datasets were pooled, and the majority-vote cluster label provided for each subject.

Mortality analysis was performed in each imputed dataset based on the labels given to each subject for said imputation, using those without respiratory symptoms as reference group. The most recent OLIN cohort (*n* = 6854) was excluded, as mortality register follow-up was only available for 1 year. All-cause mortality over time was plotted with a Kaplan–Meier plot. The association with each of the clusters was assessed with Cox proportional hazards model hazard ratio (HR) estimates with 95% confidence intervals (95% CI), unadjusted as well as adjusted for confounders selected using a literature review-based directed acyclic graph (DAG; Supplementary Fig. 3). The confounders adjusted for were: age (continuous variable); body mass index (BMI; continuous variable); sex; smoking status (current, former, never); cohort; occupational exposure to vapor gas, dust, and fumes (yes/no); educational level (primary, secondary, tertiary); socioeconomic status (SES) proxied by occupation; and comorbidity (proxied by a modified version of CCI [excluding asthma and COPD], and asthma and COPD individually). As mortality is commonly influenced by multiple factors, particularly over longer periods of time, we assessed effect modification through stratified analyses by sex (men, women), age (≤ 60 years, > 60 years), CCI (0, 1–2, ≥ 3), presence/absence of asthma/COPD, and follow-up time (≤ 5 years, ≤ 10 years). Cause-specific mortality was assessed as per above, but using the Fine-Gray subdistribution hazards model. The estimates were pooled using Rubin's rules [20]. Mortality analyses were performed if there were ≥ 10 events per covariate [21]. The proportionality assumption for these analyses was examined with statistical tests and visual inspection of Schoenfeld residuals (Supplementary text 3). Descriptive tables were produced with categorical variables reported as the proportion and compared to the other subjects/clusters with Pearson chi-square test, while continuous variables were reported as the mean and compared to the other subjects/clusters with Mann–Whitney U test.

The analyses were performed in the Python programming language 3.12.0 (Python Software Foundation) and R statistical software 4.2.3 (R Core Team). Figures were produced in R with some additional formatting in the interface design software Figma (Figma, Inc). The codes are available at https://osf.io/xtwgu.

## Results

**Fig. 2:**
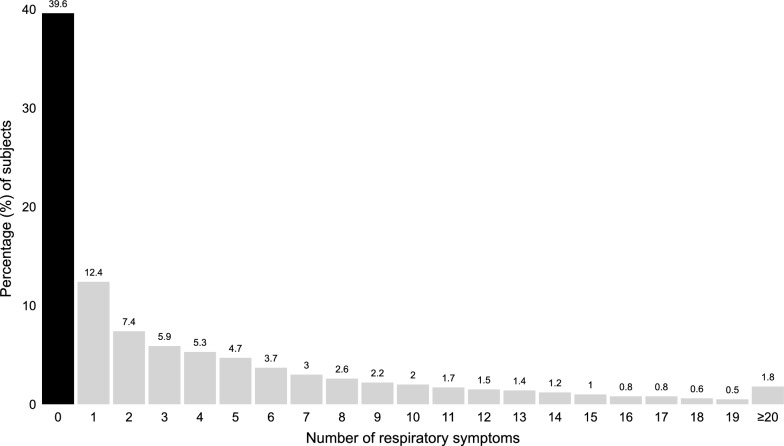
Percentage of subjects with *n* respiratory symptoms. Number of respiratory symptoms reported (x-axis) and percentage (%) of subjects reporting the respective number of respiratory symptoms (y-axis; representing the average percentage across the 100 imputed datasets). The left-most bar indicates the proportion of subjects reporting no respiratory symptoms while the right-most bar indicates the percentage of subjects reporting ≥ 20 (20–31) respiratory symptoms

The full study population (*N* = 63,060) consisted of 53% women. The average age was 48 ± 16 years. Around 10% reported physician-diagnosed asthma and almost 3% reported physician-diagnosed COPD. Fourteen percent were current smokers, and 23% reported of having smoked previously. In terms of comorbidities, 93% had CCI (excluding asthma and COPD) of 0, about 6% had CCI of 1–2, and 1% had CCI ≥ 3 (https://osf.io/sr5ny). Around 60% reported at least one respiratory symptom. Almost a third (29.5%) reported ≥ 5 respiratory symptoms, and 13.3% reported ≥ 10 respiratory symptoms (Fig. 2). The most common respiratory symptom was ever allergic rhinitis symptoms (27%), closely followed by dyspnea attack on exposure to cold air, dust, tobacco smoke, or exhaust fume (26%) (https://osf.io/sr5ny).

### Characteristics of respiratory symptom clusters

**Fig. 3:**
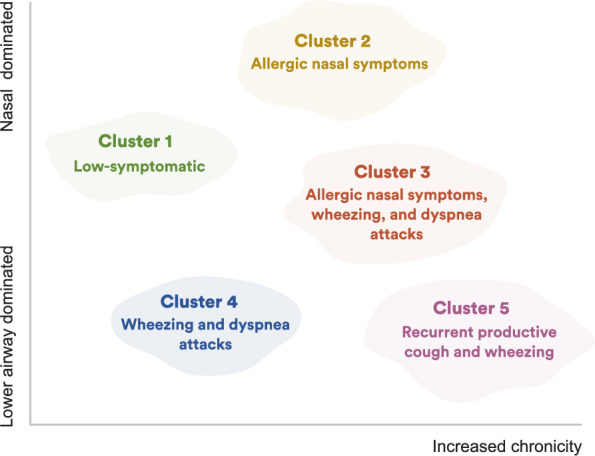
Characterization of the respiratory symptom clusters. Visualization of cluster characteristics, with the x-axis denoting chronicity of symptoms and the y-axis denoting the primary/dominating localization of respiratory symptoms

Five clusters were identified (Fig. 3, Table 1, Supplementary Table 2 and https://osf.io/2eavj for details):

- Cluster 1 ("low-symptomatic"; 30.3%): Low burden of respiratory symptoms. Most common being dyspnea attack in exposure to cold air, dust, tobacco smoke, or exhaust fume (37%). Similar proportions reported night-time symptoms, particularly waking up at night with a cough attack (30%). Chronic symptoms were uncommon, but 20% reported recurrent nasal obstruction, and a similar percentage recurrent rhinorrhea. This cluster had the lowest proportion of asthma in the family (19%) and the lowest proportion of physician-diagnosed asthma (6.2%).
- Cluster 2 ("allergic nasal symptoms"; 10.7%): Symptom pattern dominated by recurrent allergic nasal symptoms (53% with at least one symptom period of ≥ 5 days in a week and 44% with at least one such period extending for ≥ 5 weeks). Dyspnea attacks in exposure to pollen or fur was also relatively common (43%) and almost a third reported such symptoms in exposure to cold air, dust, tobacco smoke, or exhaust fume. This cluster had the lowest average BMI (24.5 ± 3.3 kg/m^2^) and the lowest proportion of physician-diagnosed COPD (1%).
- Cluster 3 ("allergic nasal symptoms, wheezing, and dyspnea attacks"; 4.7%): Cluster with dyspnea attacks (≥ 74% to all triggers except painkillers), wheezing (any wheezing in the past year: 94%, recurrent symptoms: 57%), and allergic nasal symptoms (all subjects reported any allergic rhinitis symptoms in the past year). A notable proportion (52%) also reported recurrent nasal obstruction, and 44% symptoms persisting for ≥ 13 weeks in the past year. This cluster had the highest proportion of women (62%). Around half reported family history of asthma, and 58% had been diagnosed with asthma. Finally, this cluster had the highest proportion of eczema/skin allergy ever (75%). Eighteen percent were current smokers, around 10% had physician-diagnosed COPD, and around 2% had register records for specialist cardiovascular care (Supplementary Table 2).
- Cluster 4 ("wheezing and dyspnea attacks"; 6.6%): Wheezing was a dominant symptom (100% reported wheezing in the past year and over half had recurrent wheezing). Night-time respiratory symptoms affected around half, most commonly waking up with a cough attack. Dyspnea attacks were reported in relation to physical strain and exposure to cold air, dust, tobacco smoke, or exhaust fume in roughly 60%, respectively. Slightly below a third had been diagnosed with asthma. This cluster had the second-highest proportion of subjects with register records of specialist care for cardiovascular disease (3.7%; Supplementary Table 2).
- Cluster 5 ("recurrent productive cough and wheezing"; 4.1%): High proportion of productive cough (72% reporting productive cough for ≥ 3 months for ≥ 2 years). Dyspnea attacks were also common in exposure to cold air, dust, tobacco smoke, or exhaust fume (75%). This cluster also had the highest proportion of night-time respiratory symptoms (77%). The proportion of women was the lowest (51%), while the average age (54 ± 16 years) and BMI (26.7 kg/m^2^) were the highest. Asthma had been diagnosed in 28%, with the highest average age at diagnosis (33 ± 18 years). Finally, this cluster had the highest proportion of current smokers (32%), physician-diagnosed COPD (24%), and specialist care for cardiovascular disease (3.8%; Supplementary Table 2).

**Table 1:**
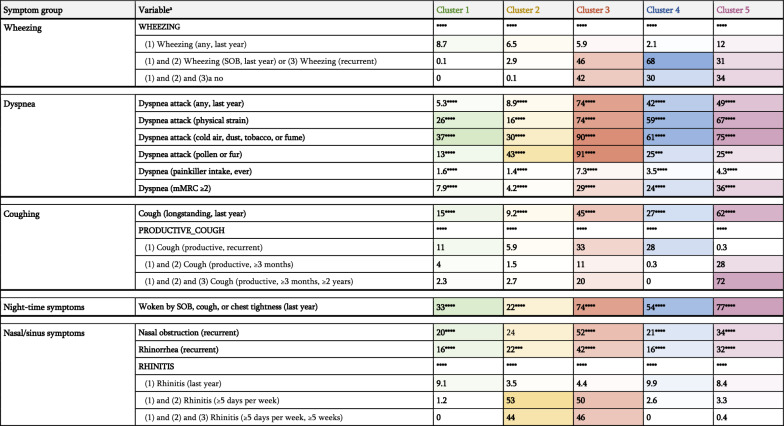
Prevalence of reported respiratory symptoms in the respiratory symptom clusters

The numeric values represent the proportion in the cluster (pooled across the 100 imputed datasets) that answered affirmatively to the corresponding question(s). The *p*-values were calculated with Chi-square test with the other clusters being the comparison group (e.g., for Cluster 4, Clusters 1–3 and Cluster 5 were grouped into one comparison group). ^a^ Denotes ordinal variables, below which are the proportions for each value. *p < 0.05. **p < 0.01. ***p < 0.001. ****p < 0.0001. mMRC: Modified Medical Research Council dyspnea scale (a score of ≥ 2 is defined as walking slower than people of the same age on ground level due to breathlessness, or having to stop for breath when walking in normal pace). SOB: shortness of breath

### Association between respiratory symptom clusters and mortality

**Fig. 4:**
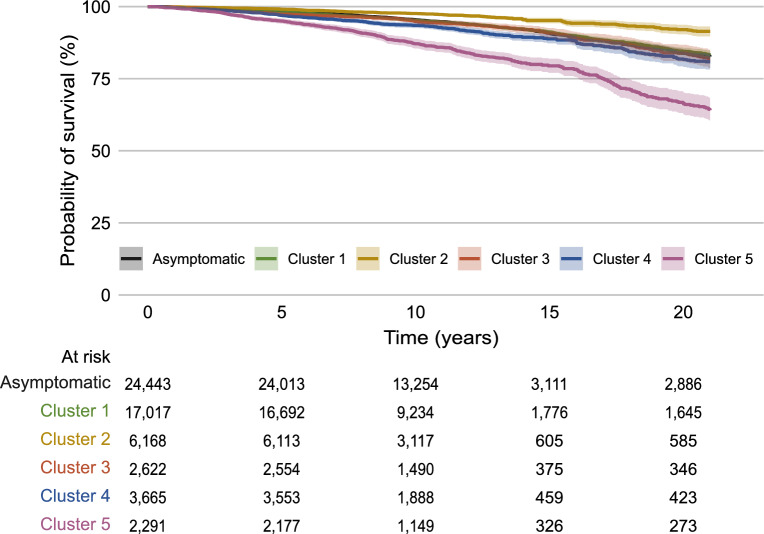
Kaplan–Meier plot for all-cause mortality. Kaplan–Meier plot of time to all-cause mortality (one line for each cluster and one line for the respiratory asymptomatic subjects, with shaded area around the line denoting the 95% confidence interval). At the bottom are the number of subjects at risk at 0, 5, 10, 15, and 20 years post-baseline (defined as the start of the year of questionnaire distribution) in each cluster (and in the respiratory asymptomatic subjects)

**Fig. 5:**
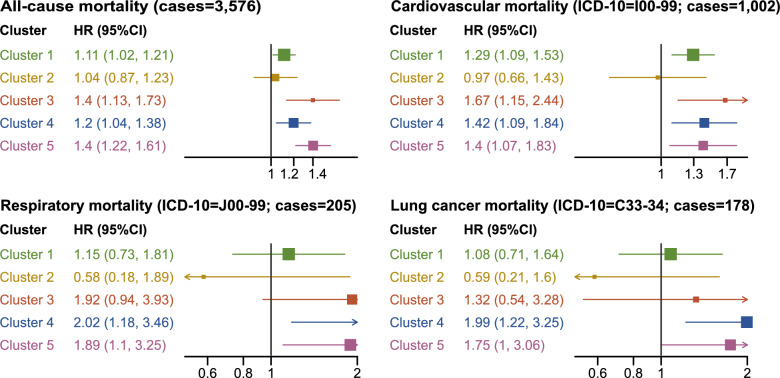
All-cause and cause-specific mortality in the respiratory symptom clusters. Clarification. Forest plots denoting the adjusted hazard ratio with 95% confidence interval for each cluster in relation to all-cause mortality (top-left panel) and cause-specific mortality. The asymptomatic subjects were used as reference. Abbreviations. HR: adjusted hazard ratio. ICD-10: International Classification of Diseases 10th Revision codes (blocks thereof, used to define the cases [outcomes]). 95% CI: 95% confidence interval

A total of 3576 deaths were recorded during the follow-up and the crude survival curves differed substantially between the groups (Fig. 4). In unadjusted analyses, all but Cluster 1 were significantly associated with all-cause mortality, the highest HR seen for Cluster 5 (HR 2.58, 95% CI 2.29–2.89). Cluster 2 was uniquely associated with lower risk (0.51, 0.43–0.59) (Supplementary Table 3). In the adjusted analyses (Fig. 5), all-cause mortality was associated with all but Cluster 2, weakest for Cluster 1 (HR 1.11, 95% CI 1.02–1.21). The strongest association was seen for Cluster 3 (1.40, 1.13–1.73) and Cluster 5 (1.40, 1.22–1.61). For cardiovascular mortality, the associations were stronger for all clusters except Cluster 2, the highest being for Cluster 3 (1.67, 1.15–2.44), followed by Cluster 4 (1.42, 1.09–1.84), and Cluster 5 (1.40, 1.07–1.83). For respiratory mortality, the association was only statistically significant for Cluster 4 (2.02, 1.18–3.46) and Cluster 5 (1.89, 1.1–3.25). Finally, for lung cancer mortality, only the association with Cluster 4 was statistically significant (1.99, 1.22–3.25). In subgroup analyses (Supplementary Figs. 4–11), the associations appeared to be somewhat stronger in men than in women. For example, Cluster 4 was associated with all-cause mortality in men (1.29, 1.07–1.54) but not in women (1.09, 0.88–1.36) (Supplementary Fig. 4). Stratifying by baseline age, the associations were typically stronger for subjects aged > 60 years than those younger, exemplified with Cluster 3 and all-cause mortality (0.99, 0.68–1.44 for subjects ≤ 60 years and 1.33, 1.01–1.75 for subjects > 60 years; Supplementary Fig. 6). The association with (cause-specific) mortality was comparable or somewhat stronger in the first five years of follow-up, except for Cluster 5, as seen with cardiovascular mortality (1.29, 0.78–2.13 for follow-up ≤ 5 years; 1.65, 1.11–2.43 for follow-up ≤ 10 years; 1.4, 1.07–1.83 for the full follow-up analysis; Supplementary Fig. 7). Subgroup analyses by CCI were limited by the small proportions of subjects with CCI 1–2 and particularly CCI ≥ 3, but in essence, the associations appeared to weaken with increased comorbidity burden, as seen for Cluster 5 and all-cause mortality (1.75, 1.51–2.02 for CCI 0; 1.49, 1.08–2.05 for CCI 1–2; 1.01, 0.58–1.75 for CCI ≥ 3). Similarly, the statistically significant associations with all-cause mortality seen in those without asthma and COPD, respectively, did not remain in diagnosed subjects (Supplementary Fig. 8).

## Discussion

We found that a broad range of respiratory symptoms are prevalent in the general adult population and that it is common to have a combination of symptoms. We identified five distinct clusters based on self-reported respiratory symptoms, and the majority lacked a diagnosis of respiratory disease. The clusters varied by chronicity, type, and frequency of symptoms. Differences were also found in background characteristics, but despite adjustment for these, as well as comorbidity burden and other confounders, the clusters were differentially associated with all-cause and cause-specific mortality.

The large sample size and representation of adults from two regions in Sweden balances generalizability with interpretability. The use of a wide range of respiratory symptoms constitutes a comprehensive representation of subjective respiratory health. In addition, the exclusive use of self-reported symptoms provides a novel approach of phenotyping, without generalizing diagnosis codes. Finally, robust statistical approaches were utilized to perform and evaluate cluster analyses, as well as adjust for potential confounders in the mortality analyses, including by intricate stratification on mortality-related covariates. Utilizing a cluster analysis approach to derive subgroups allows for data-driven segmentation that—albeit influenced by the selection of variables, clustering algorithm, and basis for choosing the optimal model—can consider much more comprehensive data than what is typically achievable through manual subtyping. However, this work also has important limitations. First, self-reported data carry risk of recall bias [22] and potential misunderstanding of questions. Second, the cross-sectional perspective limits capturing transient and progressive patterns. Third, it is possible that the findings, although representative of the Swedish adult population, are not fully generalizable to other regions, given the substantial differences in respiratory symptom patterns and by extension potential differences in underlying causes [23]. Fourth, the regression models were adjusted for a summary measure of comorbidity (CCI), based on specialist inpatient/outpatient healthcare records, which underestimated important comorbidities managed at primary care level. Such data were not available. Further, CCI was constructed within the context of hospitalized patients, it is not fully comprehensive in terms of included comorbidities, and the weighting of comorbidity importance is based on a simplified system. Nonetheless, it has been shown in validation to predict mortality quite well in diverse settings [24]. Fifth, several of the included cohorts had moderate participation rates. Responders and non-responders differed in several key parameters, such as age and sex distribution [25], although we employed a robust and well-established approach to impute missing data and minimize such bias. Sixth, although the postal surveys were largely harmonized and based on common forms, some differences were present, particularly between OLIN and WSAS surveys, in which cases the most similar questions were chosen. In a few cases where important questions were completely missing, these data were imputed based on the distribution of the remaining cohorts, which was deemed reasonable given the relative similarity between the underlying populations. Finally, certain diseases, e.g., COPD, are underdiagnosed and underreported as cause of death [26], which limits interpretation of the mortality analyses.

Around two-thirds of the full study population reported at least one respiratory symptom. Among these, we identified one low-symptomatic cluster (Cluster 1), two clusters with lower airway-dominated symptoms (Clusters 4 and 5: reflecting non-allergic asthma and COPD symptomatology, respectively; the latter differentiated primarily by chronic productive cough), one cluster with mostly allergic nasal symptoms (Cluster 2: reflecting hay fever symptomatology), and one cluster with a mixture of dyspnea, wheezing, and allergic nasal symptoms (Cluster 3: reflecting allergic asthma symptomatology). Despite partly overlapping with established phenotypes of respiratory/allergic disease, relatively low proportions reported diagnoses of these diseases: 58% and 31% reported physician-diagnosed asthma in Clusters 3 and 4, respectively, and 19% reported physician-diagnosed COPD in Cluster 5.

Several clusters were associated with increased mortality. Interestingly, even the low-symptomatic cluster (Cluster 1), was, associated, albeit marginally, with elevated risk of cardiovascular mortality, but the strongest associations were seen for clusters dominated by (chronic) lower airway symptoms. The findings for the latter are consistent with evidence of risk related to individual symptoms [6, 7, 17, 18]. Allergic rhinitis has previously been associated with lower risk of all-cause and cardiovascular mortality [27], and we similarly saw reduced or non-significant mortality risk for Cluster 2. Subgroup analyses were limited by small sample sizes, but generally indicated that the associations are somewhat stronger in men than in women. This may be due to differences in reporting patterns [28], resulting in a diluted effect of assignment to symptom-heavy clusters, but smaller spirometric lung volumes in women compared to men [29] and exposures, lifestyle, or biological mechanisms that we were unable to account for, are also possible. The stronger associations found in older subjects may reflect the differing underlying causes of respiratory symptoms in younger and older individuals, e.g., both a sedentary lifestyle/overweight and conditions more common in old age such as heart failure can cause dyspnea, nocturnal cough, and wheezing [30]. Stratified analyses based on asthma/COPD were difficult to evaluate given the small sample sizes. However, the diminishing association with higher comorbidity burden indicates that underlying conditions indeed play a substantial role in the elevated risk of mortality seen. This is also indicated by Clusters 3–5 having both the strongest associations with the mortality outcomes and the highest proportions of cardiovascular morbidity, COPD, obesity, and smoking. Of note, however, only a small minority in these clusters had comorbidities that may indicate a causal pathway with mortality, e.g., COPD. Even considering the underestimation of mild/moderate disease not covered by specialist care data, our findings indicate that respiratory symptom patterns constitute a relevant health concern. While the data presented in this paper are insufficient for clinical implementation, they constitute an important steppingstone in understanding how respiratory health from a broader perspective relates to hard and clinically relevant outcomes. Further research should validate our findings, identify the most important underlying pathological mechanisms/diseases, and, using white-box/explainable clustering algorithms, distill concise and actionable sets of symptoms/thresholds, for further investigation, treatment, and/or preventive measures. Finally, the somewhat diluted association with length of follow-up is reasonable as respiratory symptom patterns are likely dynamic over time, in addition to the many other factors associated with mortality.

## Conclusions

Five distinct respiratory symptom clusters were identified using a novel machine learning technique agnostic of diagnoses and background characteristics. The clusters varied widely, from low-symptomatic to incorporating both lower and upper nasal symptoms with high chronicity. Despite partly aligning with well-established phenotypes/diagnoses, a substantial proportion lacked diagnosis of respiratory disease in even the high-symptomatic clusters. The clusters were differentially associated with all-cause and cause-specific mortality, indicating the importance of extending this work by assessing the generalizability of these clusters and elucidating actionable risk factors.

## Supplementary Information

Supplementary material 1.

Supplementary material 2.

## Data Availability

The underlying data from questionnaires and registers used in this study cannot be shared publicly due to contractual agreement with study participants and governance restrictions; however, upon reasonable request, additional analyses can be conducted after contact with the corresponding author.
