# Supplementary material for: All-cause and cause-specific mortality in respiratory symptom clusters: a population-based multicohort study
Source: Respir Res. 2025 Apr 16;26:150. doi: 10.1186/s12931-025-03224-7 (PMC12004835; doi:10.1186/s12931-025-03224-7)
Supplement: Supplementary file 1 — Supplementary material 1. [file 12931_2025_3224_MOESM1_ESM.docx]

**STROBE Statement—checklist of items that should be included in reports of observational studies**

|  | Item No. | Recommendation | Page  No. or Location | Relevant text from manuscript |
| --- | --- | --- | --- | --- |
| **Title and abstract** | 1 | (*a*) Indicate the study’s design with a commonly used term in the title or the abstract | 1 | […] population-based multicohort study |
|  |  | (*b*) Provide in the abstract an informative and balanced summary of what was done and what was found | 3 | "Participants were derived from two population-based […]"  "Five clusters were identified, partly overlapping with […]" |
| Introduction | | | |  |
| Background/rationale | 2 | Explain the scientific background and rationale for the investigation being reported | 5 | "Respiratory symptoms have been linked to impaired lung function and […]"  "However, most often, such analyses have been limited to subjects with pre-defined and diagnosed disease." |
| Objectives | 3 | State specific objectives, including any prespecified hypotheses | 5 | "The aim of the present study was to identify and characterize clusters using an ML technique based exclusively on the presence/absence of respiratory symptoms in a population-based sample of adults. Furthermore, we assessed […]" |
| Methods | | | |  |
| Study design | 4 | Present key elements of study design early in the paper | 5-6 | "Two Swedish cohorts – West Sweden Asthma Study (WSAS; *N*=42,621) from the southwestern county of Västra Götaland, and the Obstructive Lung Disease in Northern Sweden (OLIN; *N*=20,439) from the northernmost county of Norrbotten – were used […]" |
| Setting | 5 | Describe the setting, locations, and relevant dates, including periods of recruitment, exposure, follow-up, and data collection | 5-6 | "WSAS and OLIN both consist of multiple recruitment-wave population-based cohorts, in which adults living in the study areas were randomly selected. Enrolment age was 16-75 years in the WSAS cohorts and 20-74 in OLIN." |
| Participants | 6 | (*a*) *Cohort study*—Give the eligibility criteria, and the sources and methods of selection of participants. Describe methods of follow-up  *Case-control study*—Give the eligibility criteria, and the sources and methods of case ascertainment and control selection. Give the rationale for the choice of cases and controls  *Cross-sectional study*—Give the eligibility criteria, and the sources and methods of selection of participants | 5-6 | "[…] in which adults living in the study areas were randomly selected. Enrolment age was 16-75 years in the WSAS cohorts and 20-74 in OLIN." |
|  |  | (*b*) *Cohort study*—For matched studies, give matching criteria and number of exposed and unexposed  *Case-control study*—For matched studies, give matching criteria and the number of controls per case | N/A | *Not applicable, as no specific inclusion/exclusion criteria was implemented other than the chosen age range and recruitment through randomization* |
| Variables | 7 | Clearly define all outcomes, exposures, predictors, potential confounders, and effect modifiers. Give diagnostic criteria, if applicable | 6-7 | "We included only questions that did not necessitate diagnosis of any disease for an affirmative response. The questions were binary, inquiring about the presence of various symptoms, typically within […]" |
| Data sources/ measurement | 8* | For each variable of interest, give sources of data and details of methods of assessment (measurement). Describe comparability of assessment methods if there is more than one group | 6 | "These data were extracted from the postal surveys."  "Background characteristics were also obtained from the surveys […]" |
| Bias | 9 | Describe any efforts to address potential sources of bias | 6-7 | "[…] and were used for further characterization and for confounder adjustment in the mortality analyses."  "Missing data in the postal surveys, however, was imputed using multiple imputation by chained equations (MICE) with random forests (RF)." |
| Study size | 10 | Explain how the study size was arrived at | N/A (5, 7) | *All available cohorts with usable data were used*  ("Two Swedish cohorts – West Sweden Asthma Study (WSAS; *N*=42,621) from the southwestern county of Västra Götaland, and the Obstructive Lung Disease in Northern Sweden (OLIN; *N*=20,439) from the northernmost county of Norrbotten – were used in the present study."  "The most recent OLIN cohort (*n*=6,854) was excluded, as mortality register follow-up was only available for one year.") |

Continued on next page

| Quantitative variables | 11 | Explain how quantitative variables were handled in the analyses. If applicable, describe which groupings were chosen and why | 7-8 | "[…] and the mean for continuous variables, for each subject […]"  "[…] while continuous variables were reported as the mean (pooled across the imputed datasets) and compared to the other subjects/clusters with Mann-Whitney U test." |
| --- | --- | --- | --- | --- |
| Statistical methods | 12 | (*a*) Describe all statistical methods, including those used to control for confounding | 7-8 | " Cluster analysis was performed using a modified implementation of Locality-Sensitive Hashing (LSH)-*k*-representatives (LSH-*k*-prototypes), to accommodate the […]"  "All-cause mortality over time in the derived clusters and the respiratory asymptomatic subjects was plotted with a Kaplan-Meier plot. The association with each of the clusters was assessed with Cox proportional hazards model hazard ratio (HR) estimates with 95% confidence intervals (95%CI), unadjusted as well as adjusted for […]" |
|  |  | (*b*) Describe any methods used to examine subgroups and interactions | 8 | "[…] we assessed effect modification through stratified analyses by sex (men, women), age (≤60 years, >60 years), CCI (0, 1-2, ≥3), presence/absence of asthma/COPD, and follow-up time (≤5 years, ≤10 years)." |
|  |  | (*c*) Explain how missing data were addressed | 7 | "Missing data in the postal surveys, however, was imputed using multiple imputation by chained equations (MICE) with random forests (RF). One hundred datasets were generated, with 10 iterations per dataset." |
|  |  | (*d*) *Cohort study*—If applicable, explain how loss to follow-up was addressed  *Case-control study*—If applicable, explain how matching of cases and controls was addressed  *Cross-sectional study*—If applicable, describe analytical methods taking account of sampling strategy | N/A |  |
|  |  | (*e*) Describe any sensitivity analyses | N/A |  |
| Results | | | | |
| Participants | 13* | (a) Report numbers of individuals at each stage of study—eg numbers potentially eligible, examined for eligibility, confirmed eligible, included in the study, completing follow-up, and analysed | 7, Figure 1 | "The most recent OLIN cohort (*n*=6,854) was excluded, as mortality register follow-up was only available for one year." |
|  |  | (b) Give reasons for non-participation at each stage | Figure 1 |  |
|  |  | (c) Consider use of a flow diagram | Figure 1 |  |
| Descriptive data | 14* | (a) Give characteristics of study participants (eg demographic, clinical, social) and information on exposures and potential confounders | 8 | "The full study population (*N*=63,060) across the five cohorts consisted of 53% women. The average age was 48±16 years. Around 10% reported physician-diagnosed asthma and almost 3% reported physician-diagnosed COPD. Fourteen percent were […]" |
|  |  | (b) Indicate number of participants with missing data for each variable of interest | 6, Supplementary figure 1 | "Overall missingness, however, was 11.2%." |
|  |  | (c) *Cohort study*—Summarise follow-up time (eg, average and total amount) | N/A |  |
| Outcome data | 15* | *Cohort study*—Report numbers of outcome events or summary measures over time | 10, Figures 4-5 | "A total of 3,576 deaths were recorded during the follow-up […]" |
|  |  | *Case-control study—*Report numbers in each exposure category, or summary measures of exposure | N/A |  |
|  |  | *Cross-sectional study—*Report numbers of outcome events or summary measures | N/A |  |
| Main results | 16 | (*a*) Give unadjusted estimates and, if applicable, confounder-adjusted estimates and their precision (eg, 95% confidence interval). Make clear which confounders were adjusted for and why they were included | 10, Figures 4-5, Supplementary table 3 | "In unadjusted analyses, all but Cluster 1 were significantly associated with all-cause mortality, the highest hazard ratio (HR) seen for Cluster 5 (HR 2.58, 95% confidence interval [95%CI] 2.29–2.89)." |
|  |  | (*b*) Report category boundaries when continuous variables were categorized | 10 | "Subgroup analyses by CCI were limited by the small proportions of subjects with CCI 1-2 and particularly CCI ≥3, but in essence, the associations appeared to weaken with […]" |
|  |  | (*c*) If relevant, consider translating estimates of relative risk into absolute risk for a meaningful time period | N/A |  |

Continued on next page

| Other analyses | 17 | Report other analyses done—eg analyses of subgroups and interactions, and sensitivity analyses | 10, Supplementary figures 4-11 | " In subgroup analyses (Supplementary figures 4-11), the associations appeared to be somewhat stronger in men than in women. For example, Cluster 4 was associated with […]" |
| --- | --- | --- | --- | --- |
| Discussion | | | | |
| Key results | 18 | Summarise key results with reference to study objectives | 11 | "[…] we identified five distinct clusters based solely on self-reported respiratory symptoms in a sample from two population-based adult cohorts, and the majority lacked a diagnosis of respiratory disease. The clusters varied by […]" |
| Limitations | 19 | Discuss limitations of the study, taking into account sources of potential bias or imprecision. Discuss both direction and magnitude of any potential bias | 11 | "First, the phenotyping was based on self-reported symptoms, with risk of recall bias^21^ and potential misunderstanding of […]" |
| Interpretation | 20 | Give a cautious overall interpretation of results considering objectives, limitations, multiplicity of analyses, results from similar studies, and other relevant evidence | 12 | "The findings for the latter are consistent with evidence of risk related to individual symptoms.^6,7,16,17^ Allergic rhinitis has previously been associated with […]" |
| Generalisability | 21 | Discuss the generalisability (external validity) of the study results | 11 | "Third, it is possible that the findings, although representative of the Swedish adult population, are not fully generalizable to other regions, given the […]" |
| Other information | |  | | |
| Funding | 22 | Give the source of funding and the role of the funders for the present study and, if applicable, for the original study on which the present article is based | 3, 8 | "The funders of the study had no role in study design, data collection, data analysis, data interpretation, or writing of the manuscript." |

*Give information separately for cases and controls in case-control studies and, if applicable, for exposed and unexposed groups in cohort and cross-sectional studies.

**Note:** An Explanation and Elaboration article discusses each checklist item and gives methodological background and published examples of transparent reporting. The STROBE checklist is best used in conjunction with this article (freely available on the Web sites of PLoS Medicine at http://www.plosmedicine.org/, Annals of Internal Medicine at http://www.annals.org/, and Epidemiology at http://www.epidem.com/). Information on the STROBE Initiative is available at www.strobe-statement.org.
