# Supplementary material for: All-cause and cause-specific mortality in respiratory symptom clusters: a population-based multicohort study
Source: Respir Res. 2025 Apr 16;26:150. doi: 10.1186/s12931-025-03224-7 (PMC12004835; doi:10.1186/s12931-025-03224-7)
Supplement: Supplementary file 2 — Supplementary material 2. [file 12931_2025_3224_MOESM2_ESM.docx]

**Supplementary material**

**All-cause and cause-specific mortality in respiratory symptom clusters: a population-based multicohort study**

**Daniil Lisik,^1,a^ Helena Backman,^2,a^ Hannu Kankaanranta,^1,3,4^ Rani Basna,^5,1^ Linnea Hedman,^2^ Linda Ekerljung,^1^ Fredrik Nyberg,^6^ Anne Lindberg,^7^ Göran Wennergren,^8^ Eva Rönmark,^2^ Bright I. Nwaru,^1,9,b^ Lowie Vanfleteren^10,11,b^**

^1^ Krefting Research Centre, Institute of Medicine, Sahlgrenska Academy, University of Gothenburg, Gothenburg, Sweden

^2^ Department of Public Health and Clinical Medicine, Section of Sustainable Health, the OLIN unit, Umeå University, Umeå, Sweden

^3^ Tampere University Respiratory Research Group, Faculty of Medicine and Health Technology, Tampere University, Tampere, Finland

^4^ Department of Respiratory Medicine, Seinäjoki Central Hospital, Seinäjoki, Finland

^5^ Division of Geriatric Medicine, Department of Clinical Sciences in Malmö, Lund University, Malmö, Sweden

^6^ School of Public Health and Community Medicine, Institute of Medicine, Sahlgrenska Academy, University of Gothenburg, Gothenburg, Sweden

^7^ Department of Public Health and Clinical Medicine, the OLIN unit, Umeå University, Umeå, Sweden

^8^ Department of Pediatrics, Sahlgrenska Academy, University of Gothenburg, Gothenburg, Sweden

^9^ Wallenberg Centre for Molecular and Translational Medicine, University of Gothenburg, Gothenburg, Sweden

^10^ COPD Center, Department of Respiratory Medicine and Allergology, Sahlgrenska University Hospital, Gothenburg, Sweden

^11^ Department of Internal Medicine and Clinical Nutrition, Institute of Medicine, Sahlgrenska Academy, University of Gothenburg, Gothenburg, Sweden

^a^ Daniil Lisik and Helena Backman equally contributed as first authors

^b^ Bright Ibeabughichi Nwaru and Lowie Vanfleteren equally contributed as last authors

**Corresponding author**

Daniil Lisik

Krefting Research Centre, Institute of Medicine

University of Gothenburg, Gothenburg, Sweden

[daniil.lisik@gu.se](mailto:daniil.lisik@gu.se)

**ORCID iD**

| **Daniil Lisik** | 0000-0002-0220-5961 |
| --- | --- |
| **Helena Backman** | 0000-0002-0553-8067 |
| **Hannu Kankaanranta** | 0000-0001-5258-0906 |
| **Rani Basna** | 0000-0001-7510-8460 |
| **Linnea Hedman** | 0000-0002-1630-3167 |
| **Linda Ekerljung** | 0000-0001-5784-0041 |
| **Fredrik Nyberg** | 0000-0003-0892-5668 |
| **Anne Lindberg** | 0000-0002-3292-7471 |
| **Göran Wennergren** | 0000-0002-7010-7191 |
| **Eva Rönmark** | 0000-0002-2358-8754 |
| **Bright Ibeabughichi Nwaru** | 0000-0002-2876-6089 |
| **Lowie Vanfleteren** | 0000-0002-4387-4096 |

**Contents**

[Supplementary figures 3](#_Toc170649368)

[Supplementary figure 1. Missingness in data 3](#_Toc170649369)

[Supplementary figure 2. Correlation matrix for the cluster variables 4](#_Toc170649370)

[Supplementary figure 3. Directed acyclic graph (DAG) 5](#_Toc170649371)

[Supplementary figure 4. All-cause mortality by sex and follow-up time 6](#_Toc170649372)

[Supplementary figure 5. Cardiovascular mortality by sex and follow-up time 7](#_Toc170649373)

[Supplementary figure 6. All-cause mortality by age and follow-up time 8](#_Toc170649374)

[Supplementary figure 7. Cardiovascular mortality by age and follow-up time 9](#_Toc170649375)

[Supplementary figure 8. All-cause mortality by comorbidity 10](#_Toc170649376)

[Supplementary figure 9. Cardiovascular mortality by comorbidity 11](#_Toc170649377)

[Supplementary figure 10. Respiratory mortality by comorbidity 12](#_Toc170649378)

[Supplementary figure 11. Lung cancer mortality by comorbidity 13](#_Toc170649379)

[Supplementary figure 12. Total within-cluster distance 14](#_Toc170649380)

[Supplementary tables 15](#_Toc170649381)

[Supplementary table 1. Definitions of cause-specific mortality 15](#_Toc170649382)

[Supplementary table 2. Background characteristics by cluster 16](#_Toc170649383)

[Supplementary table 3. Unadjusted all-cause mortality analysis 21](#_Toc170649384)

[Supplementary texts 22](#_Toc170649385)

[Supplementary text 1. Imputation 22](#_Toc170649386)

[Supplementary text 2. Cluster analysis 24](#_Toc170649387)

[Supplementary text 3. Mortality analysis proportionality assumption 26](#_Toc170649388)

[Supplementary references 27](#_Toc170649389)

# Supplementary figures


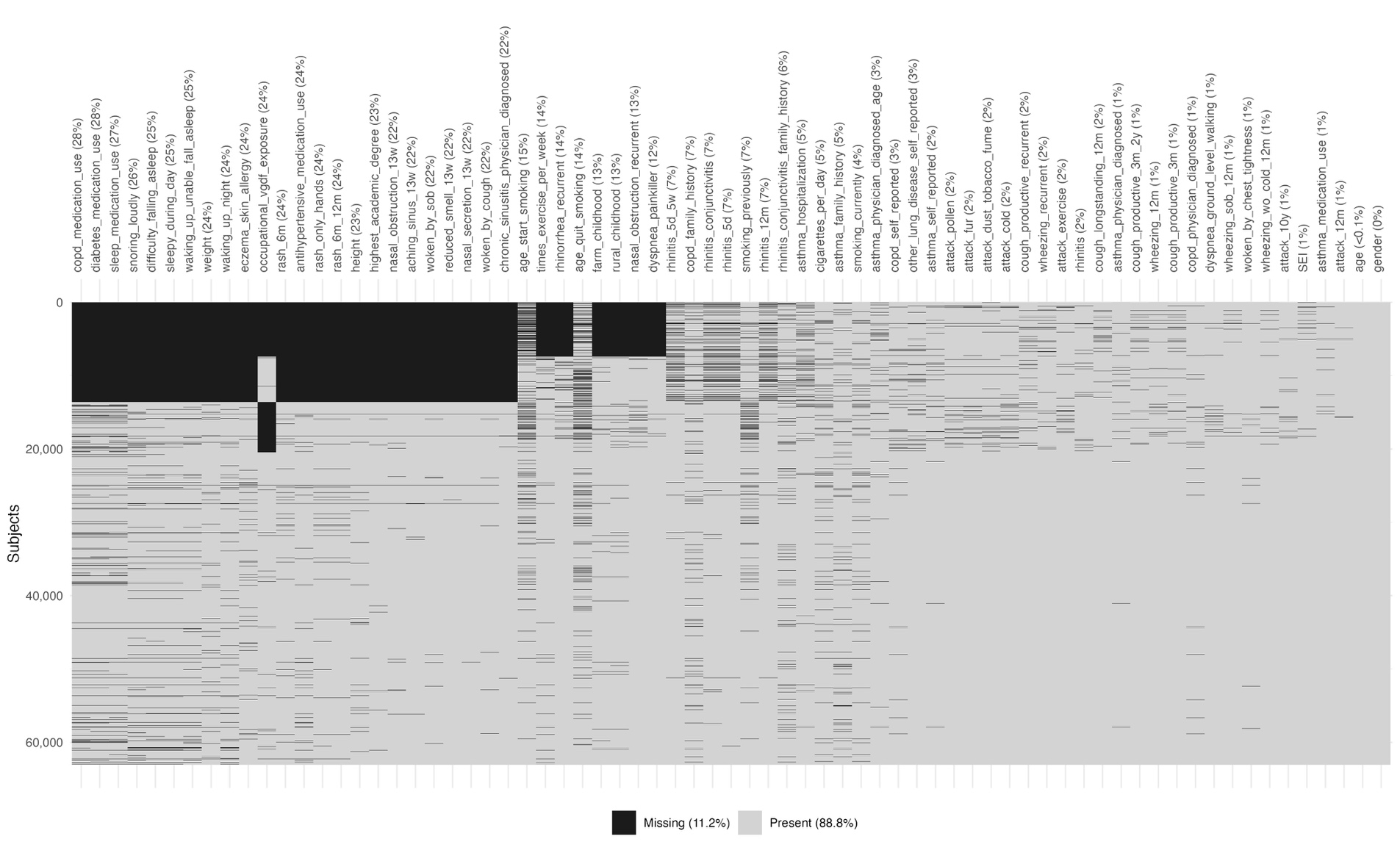


## Supplementary figure 1. Missingness in data

Visualization of missingness (each vertical level: one subject) by individual variables, sorted by decreasing missingness (left to right).


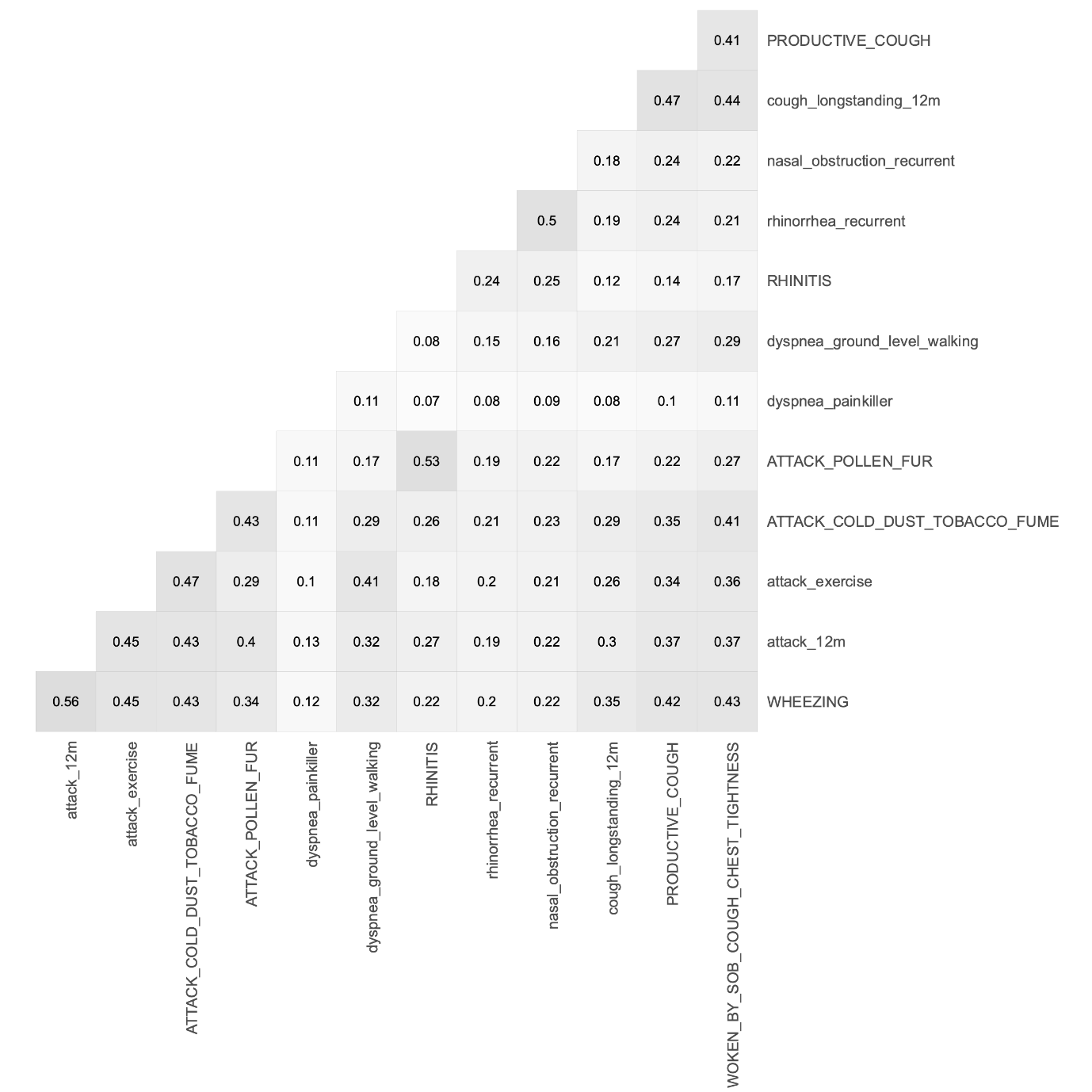


## Supplementary figure 2. Correlation matrix for the cluster variables

Spearman's rank correlation coefficient (*ρ*) between the variables selected for cluster analysis after feature engineering/selection.


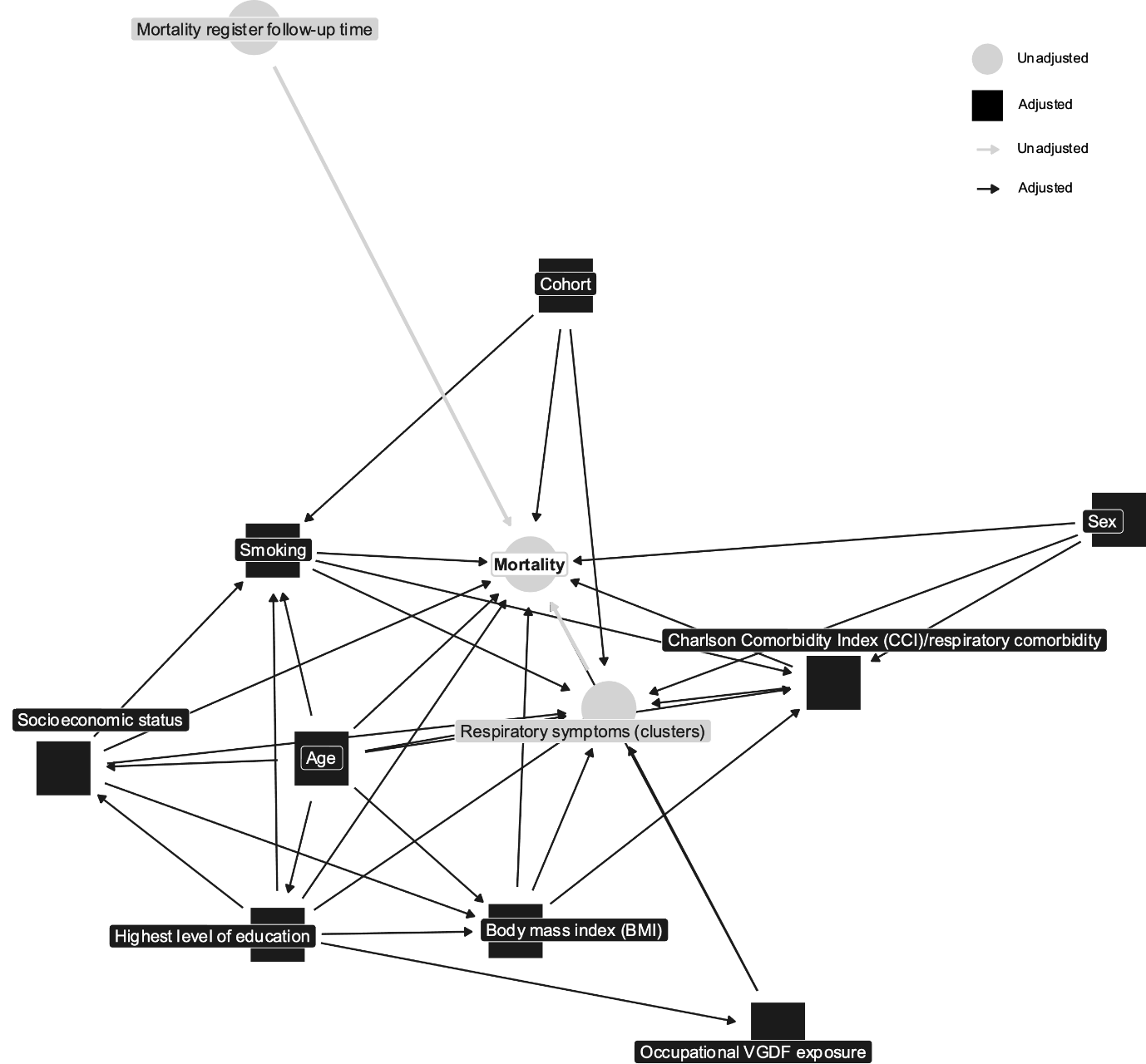


## Supplementary figure 3. Directed acyclic graph (DAG)

**Clarifications.** The variables not adjusted for are illustrated as gray circular nodes: respiratory symptoms (proxied by patterns thereof, through respiratory symptom cluster labels), the outcome (mortality), and mortality register follow-up time. Potential confounders are denoted as black square nodes. The directed edges (arrows) denote the causal effect (with direction) between two variables. Black edges denote the paths (confounders) that are adjusted for (as per the "backdoor" criterion), while the black edge denote the unadjusted path, for estimating a causal effect of the exposure on the outcome. **Abbreviations.** VGDF: vapor, gas, dust, and fumes.


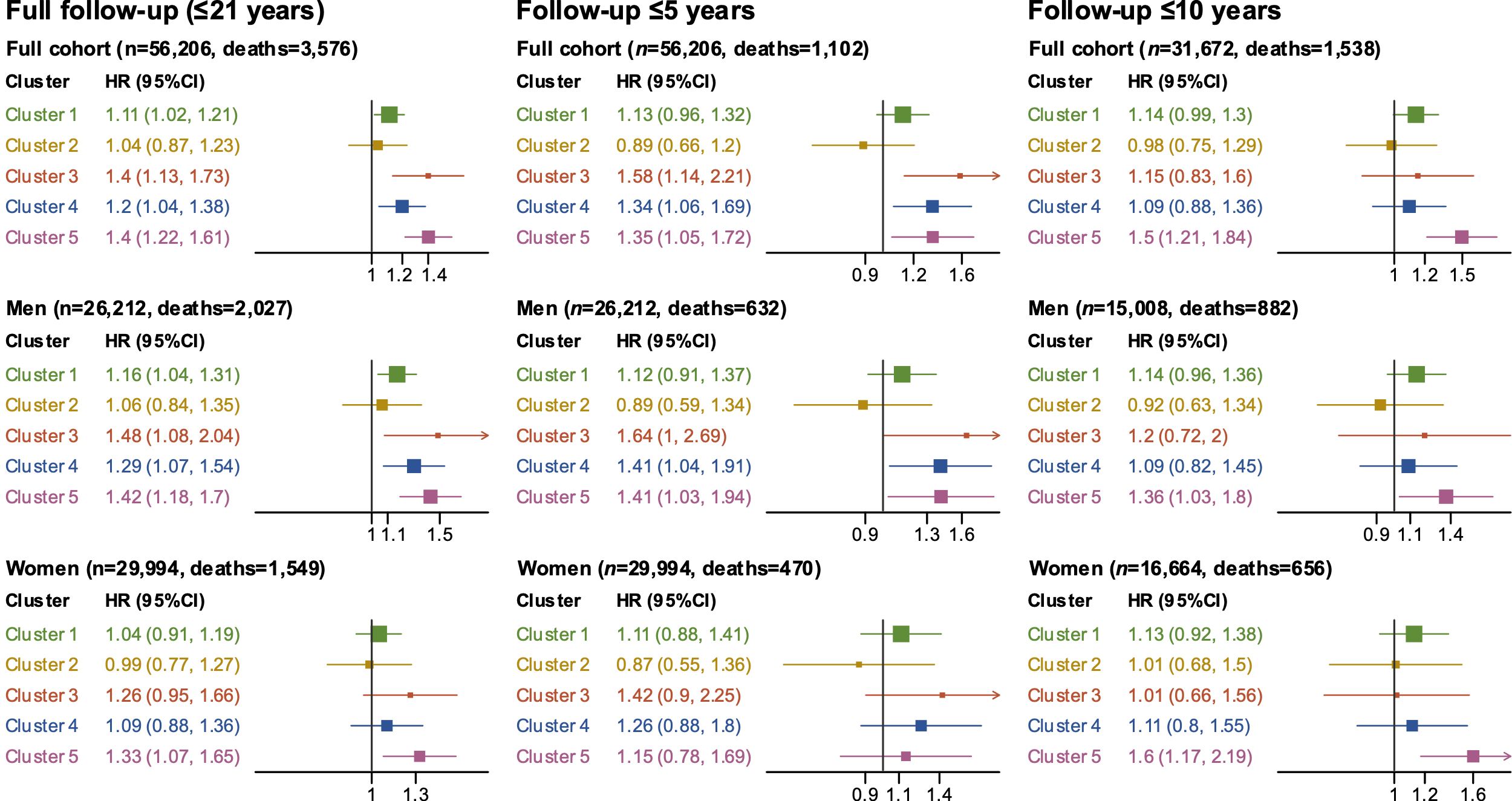


## Supplementary figure 4. All-cause mortality by sex and follow-up time

**Clarifications.** Adjusted hazard ratio with corresponding 95% confidence interval for each cluster (reference: asymptomatic subjects) in all subjects (top row) and by sex (second and third row). In the left column, the full follow-up data was used. In the center column, only the first 5 years of follow-up was taken into consideration. Subjects surviving beyond this point were right-censored. In the right column, the same principle applies, but for the first 10 years of follow-up. In addition, one cohort (WSAS-II-2016, which had only 5 years of follow-up, was excluded). **Abbreviations.** HR: adjusted hazard ratio. *n*: number of subjects at baseline. 95%CI: 95% confidence interval.


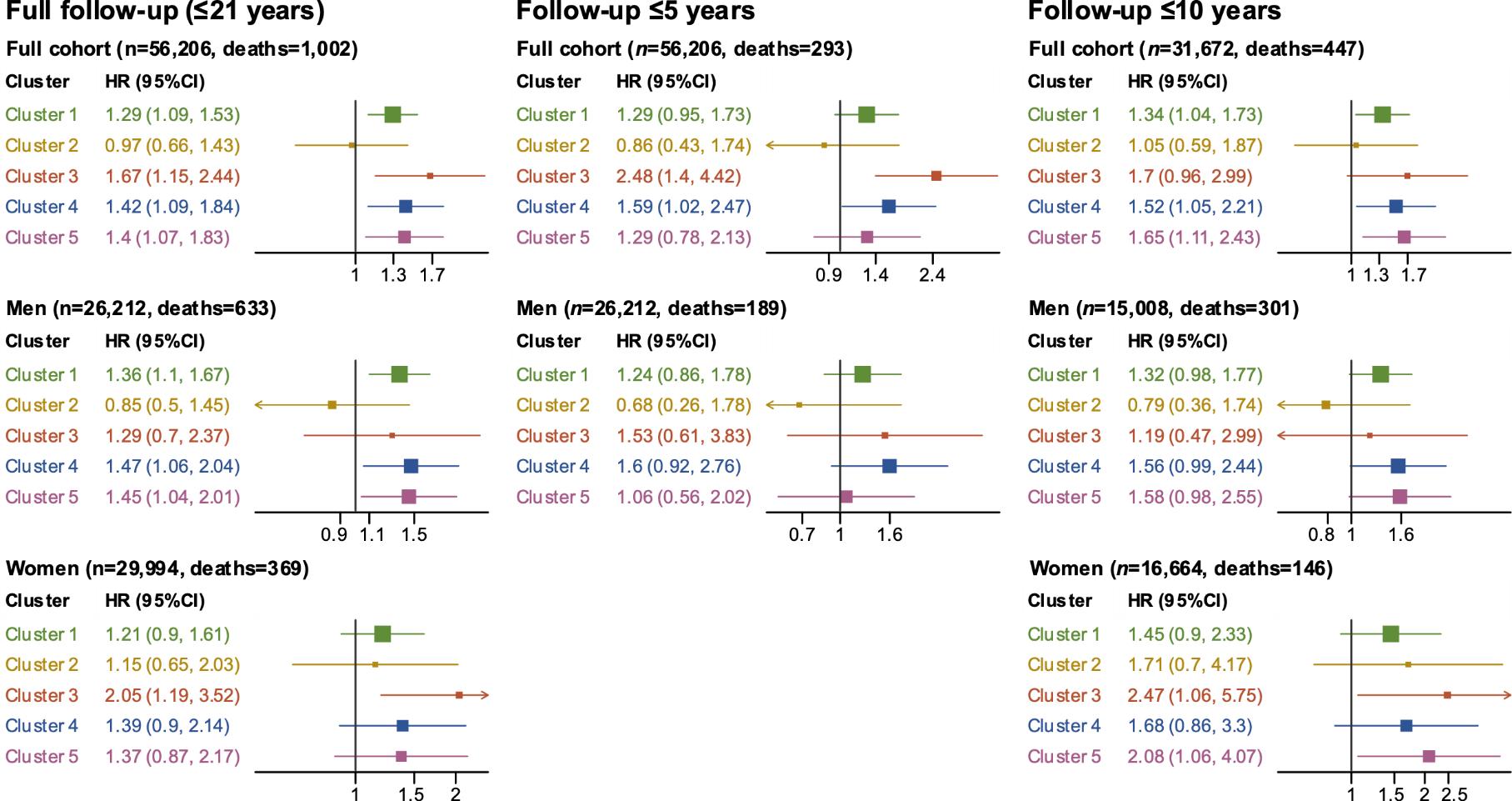


## Supplementary figure 5. Cardiovascular mortality by sex and follow-up time

**Clarifications.** Adjusted hazard ratio for cardiovascular mortality (International Statistical Classification of Diseases [ICD]-10 block I00-I99) with corresponding 95% confidence interval for each cluster (reference: asymptomatic subjects) in all subjects (top row) and by sex (second and third row). In the left column, the full follow-up data was used. In the center column, only the first 5 years of follow-up was taken into consideration. Subjects surviving beyond this point were right-censored. In the right column, the same principle applies, but for the first 10 years of follow-up. In addition, one cohort (WSAS-II-2016, which had only 5 years of follow-up, was excluded). **Abbreviations.** HR: adjusted hazard ratio. *n*: number of subjects at baseline. 95%CI: 95% confidence interval.


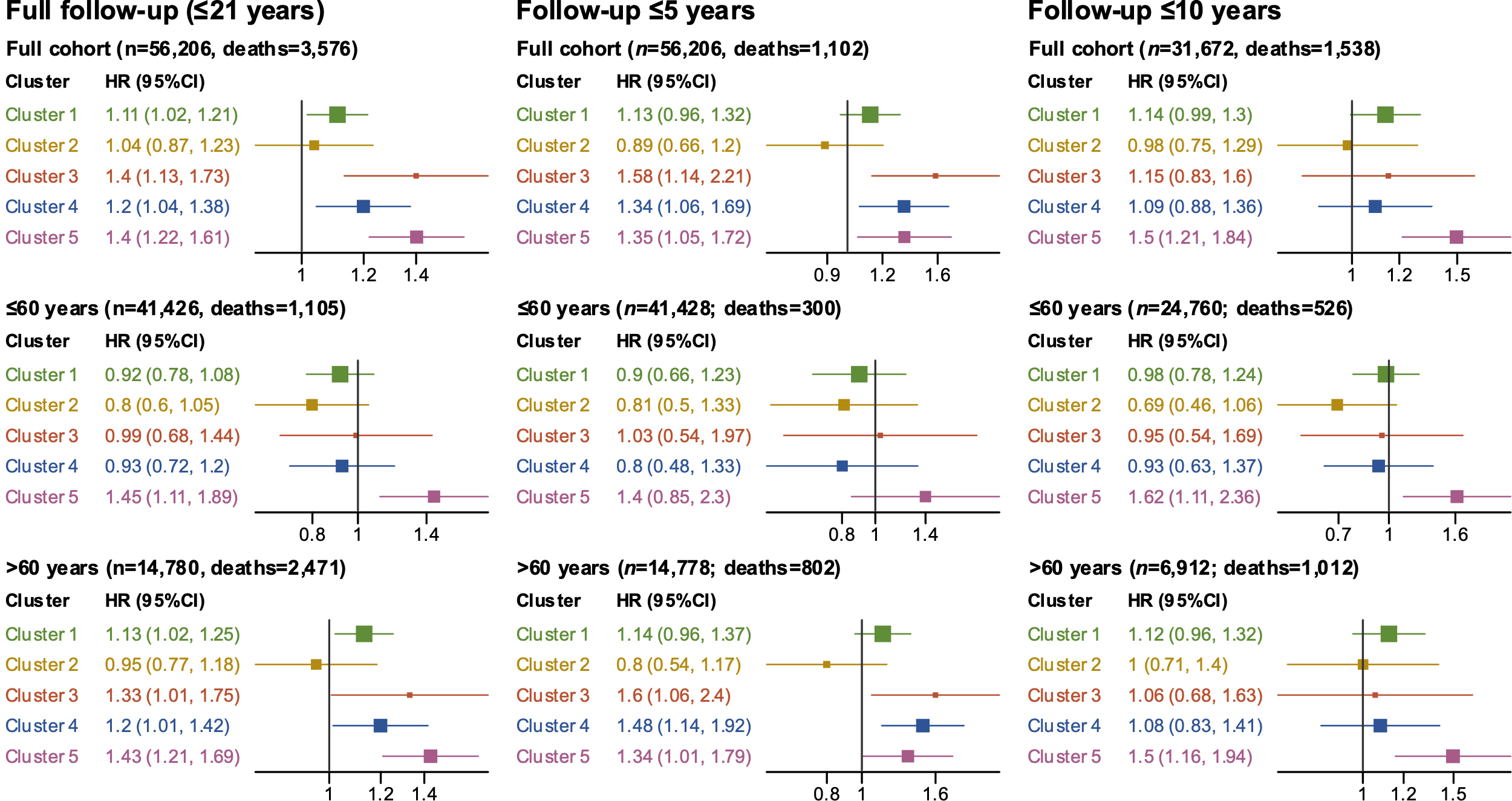


## Supplementary figure 6. All-cause mortality by age and follow-up time

**Clarifications.** Adjusted hazard ratio with corresponding 95% confidence interval for each cluster (reference: asymptomatic subjects) in all subjects (top row) and by age (second and third row). In the left column, the full follow-up data was used. In the center column, only the first 5 years of follow-up was taken into consideration. Subjects surviving beyond this point were right-censored. In the right column, the same principle applies, but for the first 10 years of follow-up. In addition, one cohort (WSAS-II-2016, which had only 5 years of follow-up, was excluded). **Abbreviations.** HR: adjusted hazard ratio. *n*: number of subjects at baseline. 95%CI: 95% confidence interval.


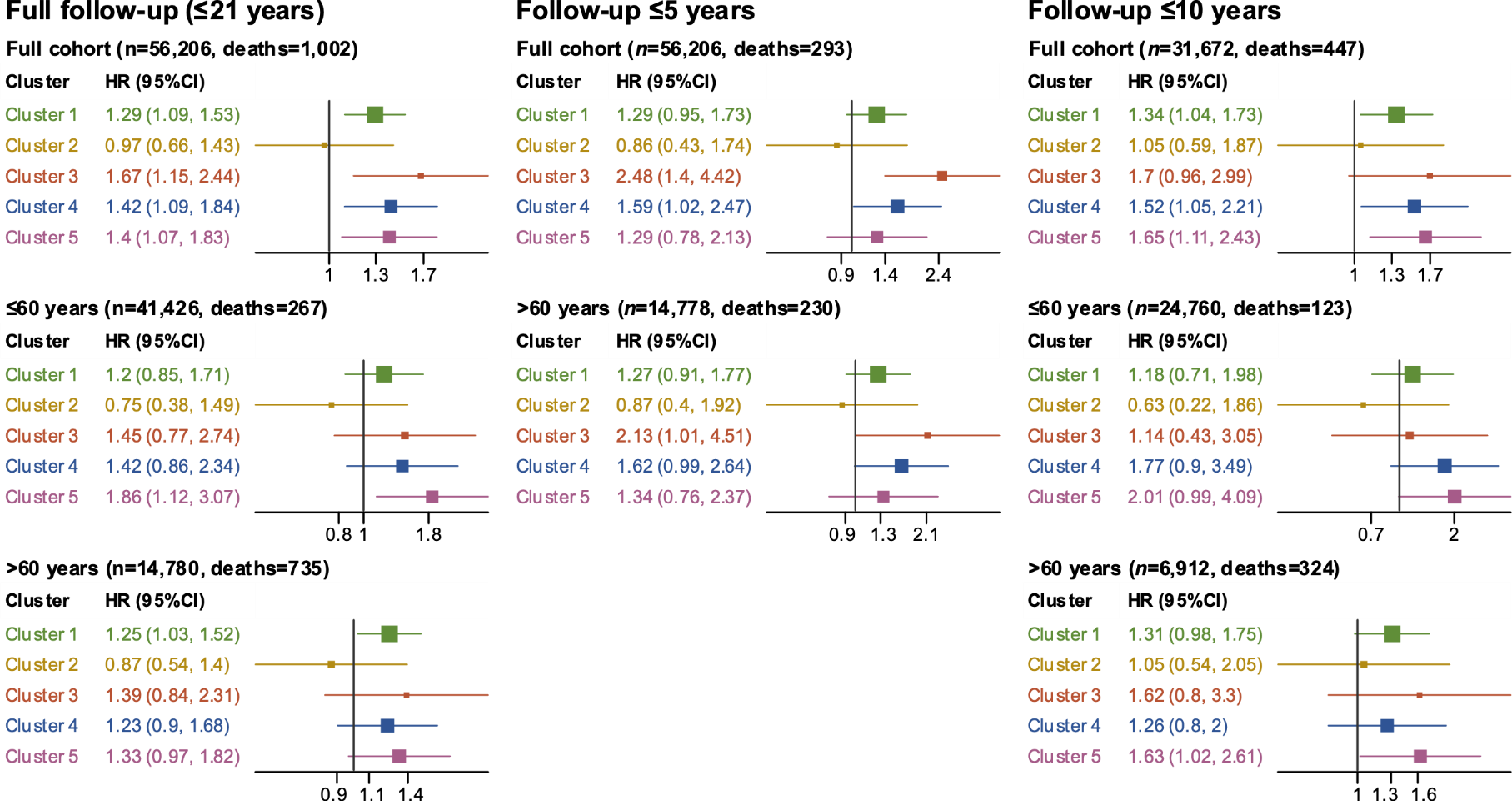


## Supplementary figure 7. Cardiovascular mortality by age and follow-up time

**Clarifications.** Adjusted hazard ratio of cardiovascular mortality (International Statistical Classification of Diseases [ICD]-10 block I00-I99) with corresponding 95% confidence interval for each cluster (reference: asymptomatic subjects) in all subjects (top row) and by age (second and third row). In the left column, the full follow-up data was used. In the center column, only the first 5 years of follow-up was taken into consideration. Subjects surviving beyond this point were right-censored. In the right column, the same principle applies, but for the first 10 years of follow-up. In addition, one cohort (WSAS-II-2016, which had only 5 years of follow-up, was excluded). **Abbreviations.** HR: adjusted hazard ratio. *n*: number of subjects at baseline. 95%CI: 95% confidence interval.


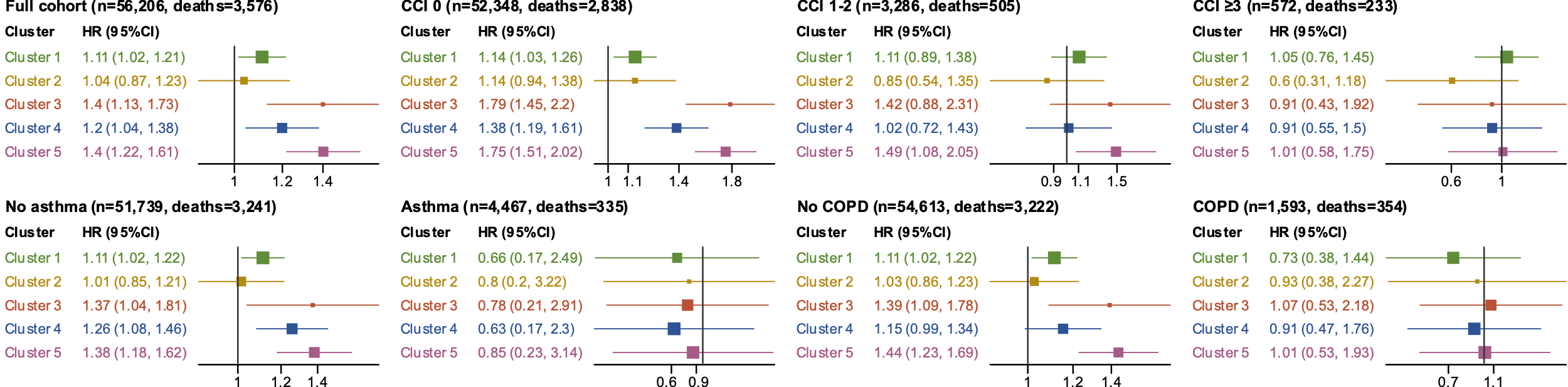


## Supplementary figure 8. All-cause mortality by comorbidity

**Clarifications.** Adjusted hazard ratio with corresponding 95% confidence interval for each cluster (reference: asymptomatic subjects) in all subjects and by overall comorbidity burden (proxied by Charlson comorbidity index) and presence/absence of physician-diagnosed asthma and chronic obstructive pulmonary disease, respectively. **Abbreviations.** CCI: Charlson comorbidity index. COPD: chronic obstructive pulmonary disease. HR: adjusted hazard ratio. *n*: number of subjects at baseline. 95%CI: 95% confidence interval.


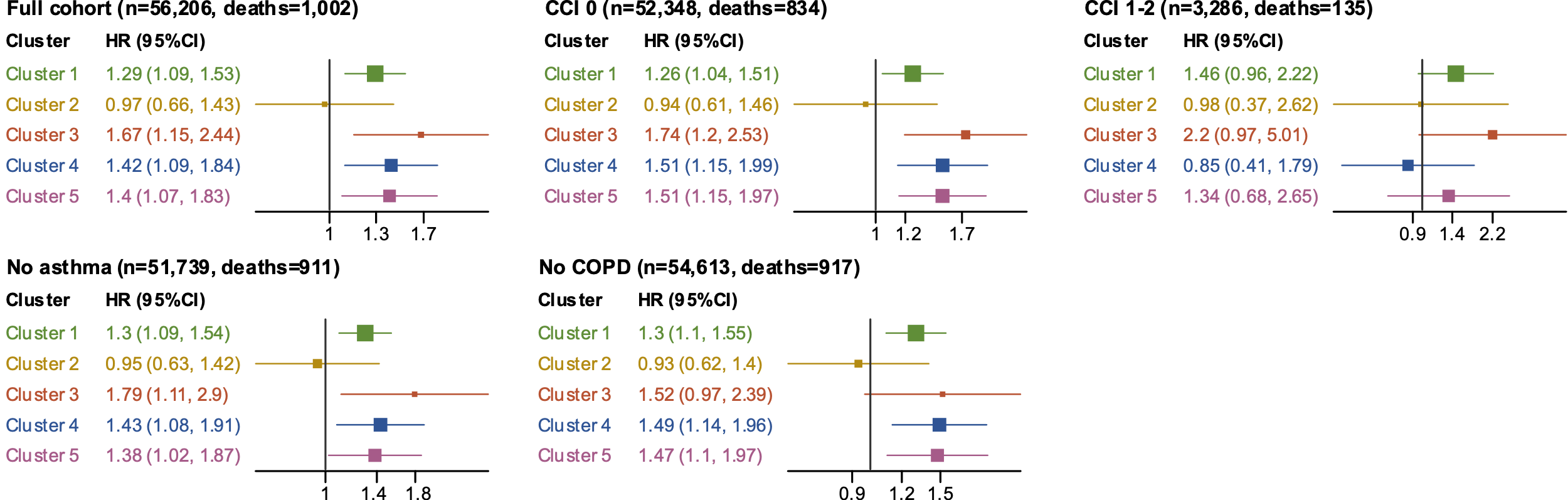


## Supplementary figure 9. Cardiovascular mortality by comorbidity

**Clarifications.** Adjusted hazard ratio for cardiovascular mortality (International Statistical Classification of Diseases [ICD]-10 block I00-I99) with corresponding 95% confidence interval for each cluster (reference: asymptomatic subjects) in all subjects and by overall comorbidity burden (proxied by Charlson comorbidity index) and absence of physician-diagnosed asthma and chronic obstructive pulmonary disease, respectively. **Abbreviations.** CCI: Charlson Comorbidity Index. COPD: chronic obstructive pulmonary disease. HR: adjusted hazard ratio. *n*: number of subjects at baseline. 95%CI: 95% confidence interval.


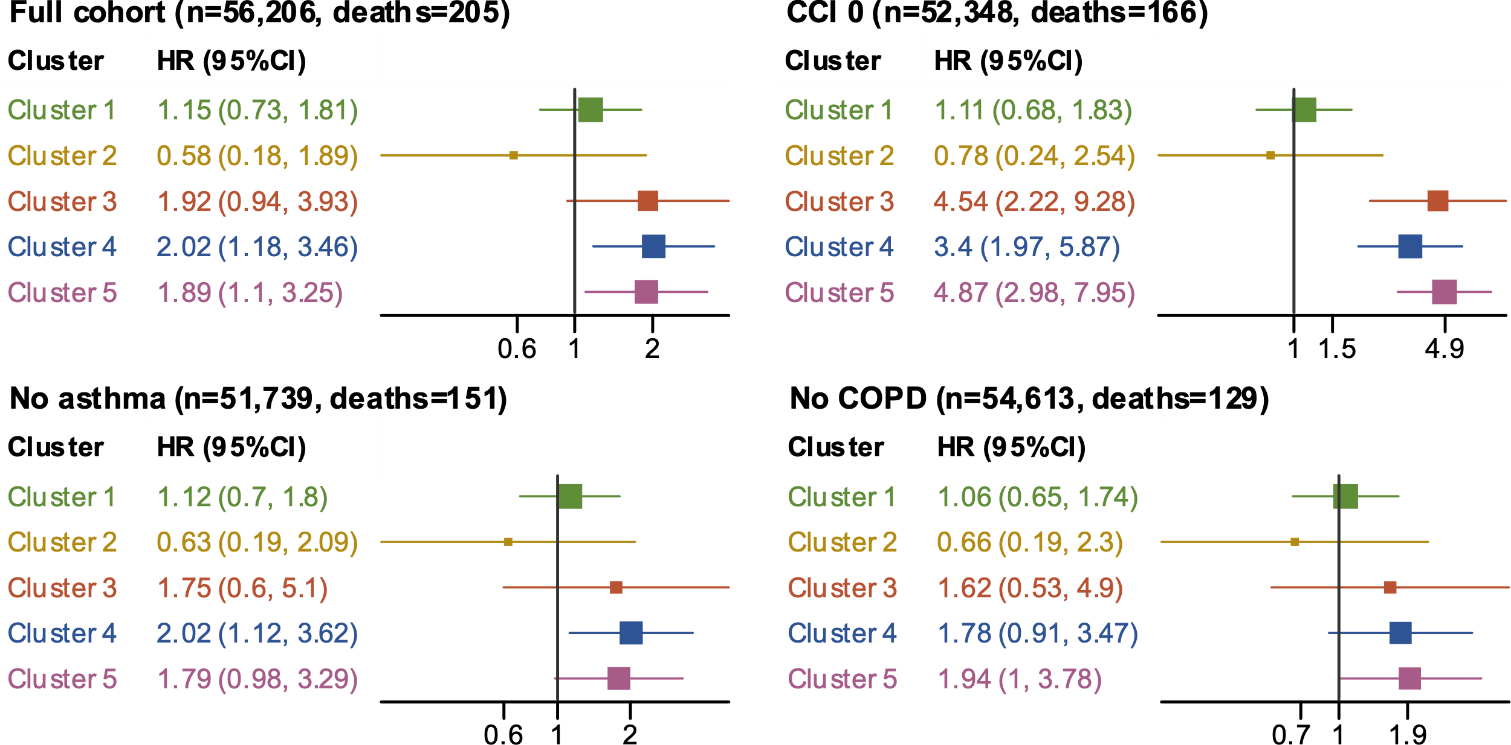


## Supplementary figure 10. Respiratory mortality by comorbidity

**Clarifications.** Adjusted hazard ratio for respiratory mortality (International Statistical Classification of Diseases [ICD]-10 block J00-J99) with corresponding 95% confidence interval for each cluster (reference: asymptomatic subjects) in all subjects and by overall comorbidity burden (proxied by Charlson comorbidity index) and absence of physician-diagnosed asthma and chronic obstructive pulmonary disease, respectively. **Abbreviations.** CCI: Charlson comorbidity index. COPD: chronic obstructive pulmonary disease. HR: adjusted hazard ratio. *n*: number of subjects at baseline. 95%CI: 95% confidence interval.


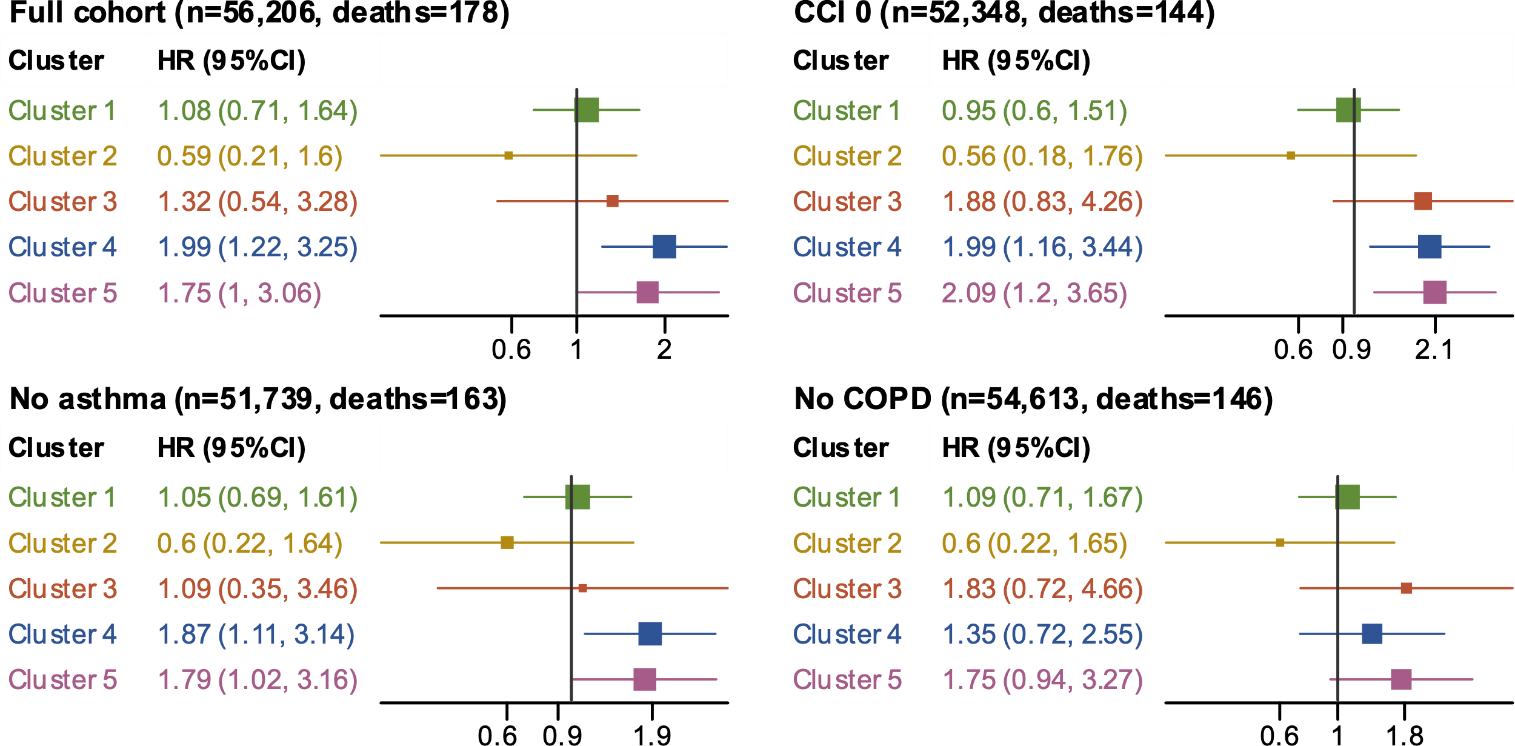


## Supplementary figure 11. Lung cancer mortality by comorbidity

**Clarifications.** Adjusted hazard ratio for lung cancer mortality (International Statistical Classification of Diseases [ICD]-10 codes C33-C34) with corresponding 95% confidence interval for each cluster (reference: asymptomatic subjects) in all subjects and by overall comorbidity burden (proxied by Charlson comorbidity index) and absence of physician-diagnosed asthma and chronic obstructive pulmonary disease, respectively. **Abbreviations.** CCI: Charlson comorbidity index. COPD: chronic obstructive pulmonary disease. HR: adjusted hazard ratio. *n*: number of subjects at baseline. 95%CI: 95% confidence interval.


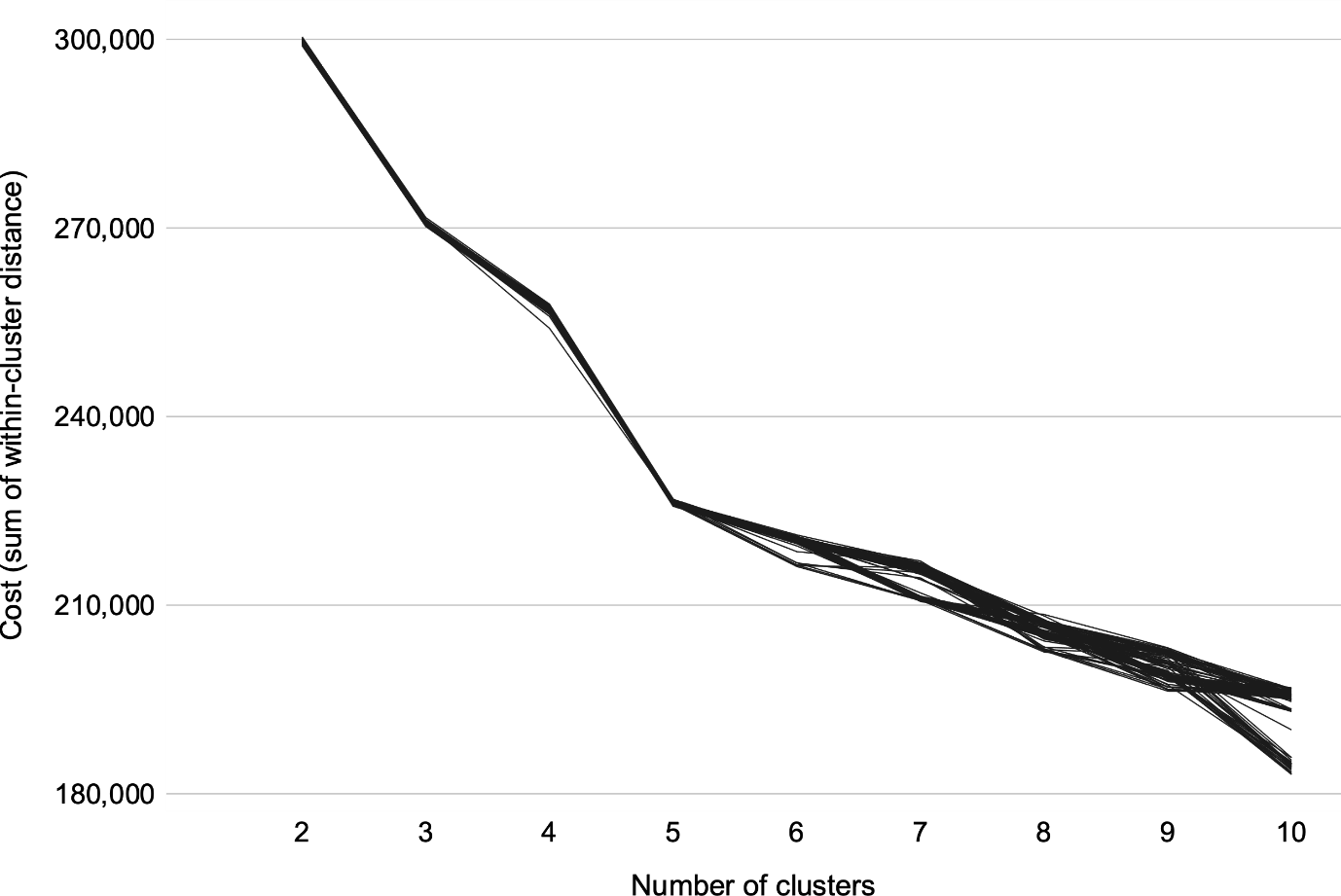


## Supplementary figure 12. Total within-cluster distance

Scree plot for total within-cluster distance (y-axis) for each number of clusters (x-axis), averaged across the 100 imputed datasets. The "elbow" in the plot (i.e., the x-value after which the rate of decrease in y-value substantially diminishes) indicates a candidate for suitable number of clusters.

# Supplementary tables

## Supplementary table 1. Definitions of cause-specific mortality

| **Outcome** | **ICD-10 code range(s)** | **Reference of utilized ICD-10 codes^a^** |
| --- | --- | --- |
| **Cardiovascular mortality** | - **I00-I99** *(diseases of the circulatory system)* | <https://doi.org/10.1038%2Fs41598-021-86407-8> |
| **Respiratory mortality** | - **J00-J99** *(diseases of the respiratory system)* | <https://doi.org/10.1016/j.rmed.2021.106518> |
| **Lung cancer mortality** | - **C33** *(malignant neoplasms of trachea)* - **C34** *(malignant neoplasms of bronchus and lung)* | <https://doi.org/10.3389%2Ffonc.2020.602397> |

**Abbreviations.** ICD-10: International Classification of Diseases, 10^th^ Revision. **Symbols.** ^a^ Reference to study in which identical/very similar definitions (in terms of utilized ICD-10 codes) of the corresponding outcome (cause-specific mortality) were used.

## Supplementary table 2. Background characteristics by cluster

| **Cluster name, *n*, and % of full study population →**  **Variable (group) ↓** | **Asymptomatic subjects**  *n* = 27,403 (43.5%) | **Cluster 1**  **Low-symptomatic**  *n* = 19,136 (30.3%) | **Cluster 2**  **Allergic nasal symptoms**  *n* = 6,755 (10.7%) | **Cluster 3**  **Allergic nasal symptoms, wheezing, and dyspnea attacks**  *n* = 2,985 (4.7%) | **Cluster 4**  **Wheezing and dyspnea attacks**  *n* = 4,172 (6.6%) | **Cluster 5**  **Recurrent productive cough and wheezing**  *n* = 2,609 (4.1%) |
| --- | --- | --- | --- | --- | --- | --- |
| **General** | | | | | | |
| **Age (years)** | **50 ± 16****** | **47 ± 17****** | **44 ± 15****** | **44 ± 15****** | **49 ± 16**** | **54 ± 16****** |
| **Gender (women)** | **51****** | **55****** | 53 | **62****** | **56***** | **51*** |
| **Body mass index (BMI)** | **25.0 ± 3.2****** | 25.3 ± 3.6 | **24.8 ± 3.3****** | **26.4 ± 3.8****** | **26.6 ± 3.9****** | **26.7 ± 3.9****** |
| **Asthma** | | | | | | |
| **Family history of asthma** | **12****** | 19 | **26****** | **46****** | **30****** | **31****** |
| **Self-reported asthma (ever)** | **1.5****** | **8****** | **15****** | **67****** | **36****** | **33****** |
| **Physician-diagnosed asthma (ever)** | **1.1****** | **6.2****** | **11****** | **58****** | **31****** | **28****** |
| **Age at asthma diagnosis** | **15 ± 15****** | **22 ± 18*** | **17 ± 13****** | **21 ± 15****** | **26 ± 18****** | **33 ± 18****** |
| **Asthma medication use (current)** | **0.4****** | **6****** | **12***** | **66****** | **36****** | **38****** |
| **Asthma hospitalization (ever)** | **0.3****** | **1.2****** | 1.7 | **10****** | **5.3****** | **4.7****** |
| **Chronic obstructive pulmonary disease (COPD)** | | | | | | |
| **Family history of COPD** | **6****** | 9.3 | 8.9 | **17****** | **15****** | **19****** |
| **Self-reported COPD (ever)** | **0.5****** | **3****** | **1.6****** | **13****** | **11****** | **24****** |
| **Physician-diagnosed COPD (ever)** | **0.2****** | **2****** | **0.9****** | **9.7****** | **8.5****** | **19****** |
| **COPD medication use (current)** | **0.1****** | **0.8****** | **0.3****** | **2.3****** | **4.4****** | **9.8****** |
| **Rhinitis and sinusitis** | | | | | | |
| **Family history of allergic rhinitis or conjunctivitis** | **16****** | **27***** | **63****** | **66****** | **33****** | **32****** |
| **Physician-diagnosed chronic sinusitis (ever)** | **0.5****** | **1.7***** | **1.9*** | **4.7****** | **2.5****** | **4.4****** |
| **Eczema and skin allergy** | | | | | | |
| **Itchy rash (≥6 months, ever)** | **5.4****** | **11***** | **16****** | **24****** | **14****** | **18****** |
| **Itchy rash (≥ 6 months, last year)** | **4.5****** | **9.3***** | **13****** | **21****** | **12****** | **16****** |
| **Itchy rash (only hands, last year)** | **0.9****** | **1.6*** | **2.1****** | **2.8****** | 1.7 | **2**** |
| **Eczema/skin allergy (ever)** | **23****** | **41****** | **59****** | **75****** | **49****** | **47****** |
| **Sleep disturbance** | | | | | | |
| **Snoring loudly (last months)** | ******** | ******** |  | ******** | ******** | ******** |
| Never/rarely | **69** | **62** | 63 | **55** | **55** | **49** |
| Less than once per week | **12** | **12** | 13 | **11** | **11** | **9.2** |
| 1-2 times per week | **9** | **10** | 10 | **10** | **11** | **11** |
| 3-5 times per week | **5** | **6.9** | 6.3 | **7.6** | **7.7** | **7.8** |
| Every or almost every night | **5.5** | **9.1** | 8.4 | **16** | **15** | **23** |
| **Difficulty falling asleep (last months)** | ******** | ******** | ******** | ******** | ******** | ******** |
| Never/rarely | **64** | **52** | **50** | **40** | **48** | **44** |
| Less than once per week | **19** | **20** | **23** | **17** | **17** | **15** |
| 1-2 times per week | **11** | **15** | **14** | **17** | **16** | **15** |
| 3-5 times per week | **4.1** | **7.4** | **7.5** | **10** | **9.4** | **11** |
| Every or almost every night | **2.5** | **5.5** | **5.6** | **16** | **9.6** | **15** |
| **Waking up at night (last months)** | ******** | ******** | ******** | ******** | ******** | ******** |
| Never/rarely | **43** | **29** | **31** | **16** | **22** | **14** |
| Less than once per week | **21** | **22** | **23** | **17** | **18** | **13** |
| 1-2 times per week | **18** | **21** | **19** | **21** | **22** | **20** |
| 3-5 times per week | **8.6** | **12** | **12** | **15** | **14** | **16** |
| Every or almost every night | **9.3** | **16** | **15** | **32** | **24** | **37** |
| **Sleepy during day (last months)** | ******** | ******** | ******** | ******** | ******** | ******** |
| Never/rarely | **33** | **17** | **14** | **5.8** | **12** | **9.9** |
| Less than once per week | **31** | **24** | **24** | **11** | **19** | **13** |
| 1-2 times per week | **23** | **33** | **34** | **27** | **33** | **30** |
| 3-5 times per week | **8.1** | **16** | **18** | **25** | **20** | **22** |
| Every or almost every night | **4.2** | **10** | **10** | **31** | **17** | **25** |
| **Waking up early, unable to fall asleep again (last months)** | ******** | ******** | ******** | ******** | ******** | ******** |
| Never/rarely | **61** | **51** | **51** | **41** | **48** | **39** |
| Less than once per week | **19** | **21** | **22** | **19** | **20** | **19** |
| 1-2 times per week | **11** | **13** | **14** | **16** | **14** | **15** |
| 3-5 times per week | **5.5** | **8.4** | **8** | **11** | **9.7** | **11** |
| Every or almost every night | **3.2** | **5.6** | **5.1** | **14** | **8.7** | **15** |
| **Sleep medication use (current)** | **3.7****** | **6.1*** | 5.4 | **10****** | **9.8** | **13****** |
| **Comorbidities** | | | | | | |
| **Self-report other lung disease (ever)** | **2.3****** | **6.6****** | **5*** | **13****** | **12****** | **17****** |
| **Antihypertensive medication use (current)** | **14****** | **15*** | **9.8****** | 14 | **21****** | **26****** |
| **Diabetes medication use (current)** | **3.2**** | **3.6*** | **2.1****** | 3.5 | **4.8****** | **5.4****** |
| **Charlson Comorbidity Index (CCI)^1^** | ****** |  | ******** |  | ******** | ******** |
| 0 | **93** | 93 | **95** | 94 | **90** | **87** |
| 1-2 | **5.8** | 6 | **4.6** | 5.5 | **8.2** | **11** |
| ≥3 | **1** | 1.1 | **0.5** | 1 | **1.5** | **2.1** |
| **Specialist care for cardiovascular disease^2^** | 1.9 | 2.1 | **1.2****** | 1.7 | **3.1***** | **3.7****** |
| **Specialist care for diabetes^3^** | 0.2 | 0.2 | **<0.1**** | 0.3 | 0.2 | **0.5***** |
| **Specialist care for liver disease^4^** | **0.2***** | 0.3 | 0.3 | 0.5 | **0.6***** | **0.7***** |
| **Specialist care for malignancy^5^** | 2.7 | 2.5 | **1.9***** | **1.9*** | **3.3**** | **3.8****** |
| **Smoking** | | | | | | |
| **Smoking status** | ******** | ******* | ******** | ******** | ******** | ******** |
| Never-smoker | **66** | **62** | **70** | **59** | **49** | **41** |
| Current smoker | **11** | **14** | **9.3** | **18** | **26** | **32** |
| Ex-smoker | **23** | **24** | **21** | **22** | **26** | **26** |
| **Cigarettes per day (current smokers)** | ******** |  | ******** |  | ******** | ******** |
| <5 | **35** | 33 | **44** | 30 | **23** | **16** |
| 5-14 | **48** | 45 | **41** | 45 | **47** | **44** |
| 15-24 | **16** | 20 | **14** | 22 | **28** | **35** |
| >24 | **1.2** | 2.2 | **0.9** | 2.9 | **1.9** | **5** |
| **Age of smoking initiation (current and ex-smokers)** | **17.4 ± 4.5****** | **16.9 ± 4.2*** | **16.8 ± 3.7*** | **16.4 ± 4.5****** | **16.7 ± 4.3****** | **16.5 ± 4.5****** |
| **Age of smoking cessation (ex-smokers)** | 36 ± 11 | 37 ± 12 | **33 ± 11****** | **33 ± 11****** | **40 ± 13****** | **43 ± 13****** |
| **Environmental exposure** | | | | | | |
| **Rural living (first five years of life)** | **38**** | 38 | **30****** | 36 | **40***** | **44****** |
| **Farm living (first five years of life)** | **14****** | **14***** | **7.2****** | **11****** | 13 | **17****** |
| **Occupational VGDF exposure** | **13****** | **21****** | **18*** | **32****** | **27****** | **37****** |
| **Socioeconomics** | | | | | | |
| **Highest academic degree** | ******** |  | ******** | ******** | ******** | ******** |
| Primary education | **15** | 14 | **7.3** | **10** | **16** | **22** |
| Secondary education | **48** | 48 | **44** | **53** | **51** | **53** |
| Tertiary education | **37** | 39 | **49** | **37** | **33** | **25** |
| **Socioeconomic status by occupation (*Socioekonomisk indelning [SEI])*** | ******** | ******** | ******** | ******** | ******** | ******** |
| Unskilled and semi-skilled workers | **19** | **19** | **15** | **22** | **22** | **25** |
| Skilled workers | **17** | **17** | **15** | **19** | **19** | **22** |
| Assistant non-manual employees | **12** | **12** | **12** | **12** | **11** | **11** |
| Intermediate non-manual employees | **22** | **22** | **26** | **20** | **18** | **15** |
| Employed and self-employed professionals, higher civil servants, and executives | **9.6** | **8.9** | **13** | **7.4** | **8.5** | **5.4** |
| Self-employed (other than professionals) | **2.6** | **2.4** | **2.1** | **2.1** | **2.9** | **2.5** |
| Students | **4.6** | **7.1** | **7.8** | **7.4** | **5.4** | **4.3** |
| Others | **13** | **12** | **8.6** | **9.8** | **12** | **15** |
| **Physical activity** | | | | | | |
| **Physical workouts per week** | ******** | ******** | ******** | ***** | ******** | ******** |
| Less than once per week or never | **23** | **27** | **21** | **27** | **33** | **40** |
| Once per week | **17** | **19** | **20** | **18** | **18** | **16** |
| 2-3 times per week | **42** | **38** | **42** | **38** | **34** | **29** |
| 4-6 times per week | **12** | **11** | **13** | **12** | **11** | **9** |
| ≥7 times per week | **5** | **4.6** | **3.8** | **5.7** | **4.7** | **6.1** |

**Clarifications.** Table structure: Characteristic values of the subjects are averaged across the 100 imputed datasets and presented as: a) mean ± standard deviation (SD) for quantitative variables; b) percentage of affirmative responses for categorical variables. The clusters correspond to groups in which the cluster label was the most common across the 100 imputed datasets. Cell background color varies based on cell value from white (0%) to gray (100%) for categorical variables. For quantitative variables, the range goes from the minimum to the maximum value across the 100 imputed datasets for the variable in question. The *p-*values were calculated with the Chi-square test (categorical variables) and the Mann Whitney U test (quantitative variables), comparing each cluster with the rest of the clusters as comparator group (e.g., for cluster 1, the *p*-values are calculated as two groups: [1] cluster 1; [2] clusters 2-5 and asymptomatic subjects). Cells with bold font indicate that the value is statistically significantly lower/higher than in the other clusters combined. Charlson comorbidity index: the score was calculated without asthma and chronic obstructive pulmonary disease (COPD). **Abbreviations.** *n*: number of subjects in the cluster. COPD: chronic obstructive pulmonary disease. **Symbols.** **^*^** *p* < 0.05. ****** *p* < 0.01. ******* *p* < 0.001. **** *p* <0.0001. ^1^ Overall comorbidity burden measure (excluding asthma and COPD) based on specialist inpatient and outpatient care records from prior to or during the year of the survey distribution. ^2^ Based on specialist inpatient and outpatient care records with International Classification of Disease (ICD) codes of congestive heart failure, cerebrovascular disease, or peripheral vascular disease. ^3^ Based on specialist inpatient and outpatient care records with ICD codes of diabetes with or without complications. ^4^ Based on specialist inpatient or outpatient care records with ICD codes of mild or severe liver disease. ^5^ Based on specialist inpatient and outpatient care records with ICD codes of malignancy or metastatic solid tumor.

## Supplementary table 3. Unadjusted all-cause mortality analysis

| **Cluster** | **HR (95%CI)** |
| --- | --- |
| **Cluster 1** | 1 (0.93–1.09) |
| **Cluster 2** | **0.51 (0.43–0.59)** |
| **Cluster 3** | **1.09 (0.94–1.27)** |
| **Cluster 4** | **1.35 (1.19–1.52)** |
| **Cluster 5** | **2.58 (2.29-2.89)** |

**Clarifications.** For each derived cluster, using respiratory asymptomatic subjects as reference, the unadjusted hazard ratio with corresponding 95% confidence interval based on the pooled cluster labels. **Abbreviations.** HR: unadjusted hazard ratio. 95%CI: 95% confidence interval.

# Supplementary texts

## Supplementary text 1. Imputation

The Swedish National Cause-of-Death Register (Swedish: *Dödsorsaksregistret*) and the Swedish National Patient Register (Swedish: *Patientregistret*) have near-complete coverage.^5,6^ For this reason, subjects with no records were assumed to have had no history of specialist outpatient/in-patient care during the defined time, and it was assumed that any data present constituted the complete data for said individual. In contrast, the postal survey data contained meaningful missingness (**Supplementary figure 1**). Three medication-related variables had missingness levels of 27-28%, closely followed by specific sleep-related questions, but overall, the missingness was roughly 11%. Nevertheless, all subjects had at least one missing variable. Most of the missing data were from cohorts for which said variable was missing altogether (<https://osf.io/jsf4k> and <https://osf.io/h48es>). Some variables also had artificially high missingness, being follow-up questions necessitating affirmative response to a previous question and otherwise be skipped (e.g., age at asthma diagnosis, which had massive missingness if not accounting for subjects that explicitly reported no asthma diagnosis).

Multiple imputation by chained equations (MICE) with random forests (RF) was utilized to impute missing respiratory symptom/background characteristic data. MICE is a powerful technique for substituting missing data and thus enabling inclusion of all subjects. Unbiased estimates and valid inference can be produced with MICE if the data are missing at random (MAR).^7^ This assumption is reasonable in epidemiological context as in the present study. In contrast, missing completely at random, is uncommon, while missing not at random (MNAR), is impossible to accurately distinguish from MAR.^8^ Thus, we assumed the data to be MAR. MICE with RF utilizes a tree-based method (random forests), in which multiple regression trees with bootstrap samples from the observed data are generated and imputations randomly drawn from.^7^ Simulation studies have found MICE with RF to be a suitable imputation technique for complex epidemiologic data.^9^ An initial imputation round with 20 iterations^10^ was performed to assess the number of iterations necessary for convergence. It was found that convergence was achieved substantially earlier. For this reason, 10 iterations was used, to ensure convergence while reducing computational cost. One hundred imputed datasets were generated, in line with common practices.^11,12^ Seeding was used for reproducibility. Overall, the imputation converged well (see folder "Imputation" at <https://osf.io/xtwgu/>). There was no meaningful difference between the non-imputed and pooled imputed data (<https://osf.io/sr5ny>). Imputation was performed with the miceRanger R package.^13^

## Supplementary text 2. Cluster analysis

Categorical data are notoriously difficult to cluster with unsupervised machine learning techniques, as such data commonly lacks useful distance measures, which continuous variables naturally possess.^14,15^ In the case of nominal/binary data, it is particularly challenging, as ordinal data at least has inherent order and, depending on the number of levels, a substantially closer interpretation to continuous data.^16^ High dimensionality of data poses additional potential issues. First, depending on algorithm, it can heavily increase computational costs for the cluster analysis.^17^ Second, higher sets of variables may cause observations to become dramatically sparse in the feature space and reduce the possibility to distinguish distinct subgroups.^18,19^

In the late 1990s, Huang introduced *k*-modes,^20,21^ an extension of the *k*-means clustering algorithm to categorical data. In the years that followed, a large set of iterations and complementary methods have been developed for clustering categorical and mixed data. In the present work, a modification of the Locality Sensitive Hashing (LSH)-*k*-representatives (LSH-*k*-prototypes), was utilized for the cluster analysis, to derive clusters based solely on the presence of absence of respiratory symptoms (i.e., categorical variables). LSH-*k*-representatives was proposed by Mau and Nuynh.^22^ LSH-*k*-representatives can accommodate large datasets of categorical data and uphold high clustering quality despite substantial performance improvements. The integral *k*-representatives algorithm tackles the potential instability arising with *k-*modes in case of equal frequency of ≥2 values in clustering variables, through the introduction of representatives in lieu of modes in *k*-models. Simultaneously, utilizing LSH (facilitating management of high-dimensional data) and distance learning dissimilarity for categorical data (DILCA; a context-based dissimilarity measure [taking into account number of levels in trajectory-defining variables, their distribution etc] for categorical data) for cluster initialization, provides efficient selection of initial clusters. To better account for the ordinal nature in some of the trajectory-defining variables (<https://osf.io/hykfz>), a modification of the algorithm was used in the present work, more specifically an implementation of LSH-*k*-prototypes, to be able to calculate distance differently for nominal (binary) variables (DILCA) and Manhattan distance for ordinal variables.^23,24^

The clustering algorithm was implemented based on a modification of the Python programming language 3.12.0 (Python Software Foundation) imputation by the first author. Seeding was used for reproducibility. The code can be found in the "Scripts" folder at <https://osf.io/xtwgu/>.

Selection of the optimal clustering solution (or, rather the optimal number of clusters), is lacking a well-established gold standard, given the many intricacies that individual datasets, clustering algorithms, and research objectives.^25,26^ In the present work, given that the data were expected to have substantial overlapping components (as the trajectory-defining variables not seldom co-occur in various combinations),^27^ metrics that evaluate both intra-cluster and inter-cluster distance to derive the optimal solution, such as Silhouette score, was not utilized. Instead, the total within-cluster distance was assessed through a scree plot. The "elbow", i.e., the x-value (number of clusters), after which the decrease of the y-value substantially diminishes, constitutes the suggested optimal number of clusters. This metric suggested the solution with five clusters across the imputed datasets (**Supplementary figure 12**). To further assess the goodness of fit in the cluster solution, randomly selected rows (subjects) were removed from the data in 10% increments and re-clustered until 50% remained. The derived clusters remained nearly identical in composition and characteristics at 50% of the full respiratory symptomatic sample (<https://osf.io/597zx>). These findings were assessed by DL, RB, and LW. The cluster solutions (2-10 clusters) were also assessed clinically by DL in consultation with LW. Based on the collective appreciation of the three above approaches, the five cluster solution was ultimately selected.

Finally, we evaluated the importance of individual respiratory symptoms by gradient boosting on decision trees (CatBoost; implemented with the catboost R package),^28^ particularly useful for categorical data. CatBoost was run based on the pooled cluster labels, with normalized output of the most important (in descending order) variables for the classification illustrated in <https://osf.io/ukpaf>, indicating that wheezing and rhinitis were most influential role, with various dyspnea variables and to some extent night-time respiratory and cough variables also differentiating the clusters.

## Supplementary text 3. Mortality analysis proportionality assumption

The proportionality assumption of the Cox proportional hazards model^29^ and the Fine-Gray subdistribution hazards model^30^ was evaluated with statistical tests and visual inspection of Schoenfeld residuals. A goodness-of-fit test based on such residuals returned a non-significant *p*-value (0.5) for the cluster covariate in the Cox proportional hazards model (<https://osf.io/s8jtp>) and no apparent asymmetrical pattern in the residuals over time. For some of the remaining covariates, similar Schoenfeld residual patterns were seen, albeit the *p*-value was significant for some. In addition, the Kaplan-Meier plot curves were approximately parallel.^31^ It must be noted, that with large sample sizes, retrieveing low *p*-values is higher.^32^ The covariates demonstrating the lowest *p-*value and most notably (albeit minimally) skewed distribution of Schoenfeld residuals over time (age and CCI) were stratified for in the subgroup analyses to account for any potential modifying effect. Similar findings as per above were found for the Fine-Gray subdistribution hazards model (<https://osf.io/39xdg>).

It is important to acknowledge that mortality associations are averaged across the follow-up time. Furthermore, the follow-up was relatively long (up to 21 years). Finally, it has been argued that statistical tests to evaluate the proportionality assumption are unnecessary given that hazards commonly do not remain constant over time in the context of medical studies. In summary, while we cannot rule out with absolute certainty that the proportionality assumption has not been violated, there is no indications that the findings are not clinically reliable or reasonable.^33^

# Supplementary references

1. Andersén H, Ilmarinen P, Honkamäki J, et al. NSAID-exacerbated respiratory disease: a population study. *ERJ Open Res*. Jan 2022;8(1)doi:10.1183/23120541.00462-2021

2. Mahler DA, Wells CK. Evaluation of Clinical Methods for Rating Dyspnea. *Chest*. 1988/03/01/ 1988;93(3):580-586. doi:<https://doi.org/10.1378/chest.93.3.580>

3. Ertan Yazar E, Niksarlioglu EY, Yigitbas B, Bayraktaroglu M. How to Utilize CAT and mMRC Scores to Assess Symptom Status of Patients with COPD in Clinical Practice? *Medeni Med J*. Jun 23 2022;37(2):173-179. doi:10.4274/MMJ.galenos.2022.06787

4. Lancel M, van Marle HJF, Van Veen MM, van Schagen AM. Disturbed Sleep in PTSD: Thinking Beyond Nightmares. *Front Psychiatry*. 2021;12:767760. doi:10.3389/fpsyt.2021.767760

5. Laugesen K, Ludvigsson JF, Schmidt M, et al. Nordic Health Registry-Based Research: A Review of Health Care Systems and Key Registries. *Clin Epidemiol*. 2021;13:533-554. doi:10.2147/clep.S314959

6. Brooke HL, Talbäck M, Hörnblad J, et al. The Swedish cause of death register. *Eur J Epidemiol*. Sep 2017;32(9):765-773. doi:10.1007/s10654-017-0316-1

7. Slade E, Naylor MG. A fair comparison of tree-based and parametric methods in multiple imputation by chained equations. *Stat Med*. Apr 15 2020;39(8):1156-1166. doi:10.1002/sim.8468

8. Perkins NJ, Cole SR, Harel O, et al. Principled Approaches to Missing Data in Epidemiologic Studies. *Am J Epidemiol*. Mar 1 2018;187(3):568-575. doi:10.1093/aje/kwx348

9. Shah AD, Bartlett JW, Carpenter J, Nicholas O, Hemingway H. Comparison of Random Forest and Parametric Imputation Models for Imputing Missing Data Using MICE: A CALIBER Study. *American Journal of Epidemiology*. 2014;179(6):764-774. doi:10.1093/aje/kwt312

10. Mera-Gaona M, Neumann U, Vargas-Canas R, López DM. Evaluating the impact of multivariate imputation by MICE in feature selection. *PLoS One*. 2021;16(7):e0254720. doi:10.1371/journal.pone.0254720

11. Pampaka M, Hutcheson G, Williams J. Handling missing data: analysis of a challenging data set using multiple imputation. *International Journal of Research & Method in Education*. 2016/01/02 2016;39(1):19-37. doi:10.1080/1743727X.2014.979146

12. Austin PC, White IR, Lee DS, van Buuren S. Missing Data in Clinical Research: A Tutorial on Multiple Imputation. *Canadian Journal of Cardiology*. 2021/09/01/ 2021;37(9):1322-1331. doi:<https://doi.org/10.1016/j.cjca.2020.11.010>

13. Wilson S. *miceRanger: Multiple Imputation by Chained Equations with Random Forests*. 2021.

14. Uddin J, Ghazali R, J HA, Shah H, Husaini NA, Zeb A. Rough set based information theoretic approach for clustering uncertain categorical data. *PLoS One*. 2022;17(5):e0265190. doi:10.1371/journal.pone.0265190

15. Ghassempour S, Girosi F, Maeder A. Clustering multivariate time series using Hidden Markov Models. *Int J Environ Res Public Health*. Mar 6 2014;11(3):2741-63. doi:10.3390/ijerph110302741

16. Robitzsch A. Why Ordinal Variables Can (Almost) Always Be Treated as Continuous Variables: Clarifying Assumptions of Robust Continuous and Ordinal Factor Analysis Estimation Methods. Perspective. *Frontiers in Education*. 2020;5

17. Kadir SN, Goodman DF, Harris KD. High-dimensional cluster analysis with the masked EM algorithm. *Neural Comput*. Nov 2014;26(11):2379-94. doi:10.1162/NECO_a_00661

18. Bellman R. Dynamic programming. *science*. 1966;153(3731):34-37.

19. Verleysen M, François D. The Curse of Dimensionality in Data Mining and Time Series Prediction. Springer Berlin Heidelberg; 2005:758-770.

20. Huang Z. A fast clustering algorithm to cluster very large categorical data sets in data mining. *Dmkd*. 1997;3(8):34-39.

21. Huang Z. Extensions to the k-Means Algorithm for Clustering Large Data Sets with Categorical Values. *Data Mining and Knowledge Discovery*. 1998/09/01 1998;2(3):283-304. doi:10.1023/A:1009769707641

22. Mau TN, Huynh V-N. An LSH-based k-representatives clustering method for large categorical data. *Neurocomputing*. 2021/11/06/ 2021;463:29-44. doi:<https://doi.org/10.1016/j.neucom.2021.08.050>

23. de Chiusole D, Spoto A, Stefanutti L. Extracting partially ordered clusters from ordinal polytomous data. *Behavior Research Methods*. 2020/04/01 2020;52(2):503-520. doi:10.3758/s13428-019-01248-8

24. Coombes CE, Liu X, Abrams ZB, Coombes KR, Brock G. Simulation-derived best practices for clustering clinical data. *J Biomed Inform*. Jun 2021;118:103788. doi:10.1016/j.jbi.2021.103788

25. Hu CW, Kornblau SM, Slater JH, Qutub AA. Progeny Clustering: A Method to Identify Biological Phenotypes. *Sci Rep*. Aug 12 2015;5:12894. doi:10.1038/srep12894

26. Liu T, Yu H, Blair RH. Stability estimation for unsupervised clustering: A review. *Wiley Interdiscip Rev Comput Stat*. Nov-Dec 2022;14(6):e1575. doi:10.1002/wics.1575

27. Amaral R, Bousquet J, Pereira AM, et al. Disentangling the heterogeneity of allergic respiratory diseases by latent class analysis reveals novel phenotypes. *Allergy*. 2019/04/01 2019;74(4):698-708. doi:<https://doi.org/10.1111/all.13670>

28. Dorogush AV, Ershov V, Gulin A. CatBoost: gradient boosting with categorical features support. *arXiv preprint arXiv:181011363*. 2018;

29. Abd ElHafeez S, D'Arrigo G, Leonardis D, Fusaro M, Tripepi G, Roumeliotis S. Methods to Analyze Time-to-Event Data: The Cox Regression Analysis. *Oxid Med Cell Longev*. 2021;2021:1302811. doi:10.1155/2021/1302811

30. Li J, Scheike TH, Zhang MJ. Checking Fine and Gray subdistribution hazards model with cumulative sums of residuals. *Lifetime Data Anal*. Apr 2015;21(2):197-217. doi:10.1007/s10985-014-9313-9

31. Piovani D, Nikolopoulos GK, Bonovas S. Pitfalls and perils of survival analysis under incorrect assumptions: the case of COVID-19 data. *Biomedica*. Oct 15 2021;41(Sp. 2):21-28. Escollos y peligros del análisis de supervivencia bajo supuestos incorrectos: el caso de los datos de COVID-19. doi:10.7705/biomedica.5987

32. Forstmeier W, Wagenmakers E-J, Parker TH. Detecting and avoiding likely false-positive findings – a practical guide. <https://doi.org/10.1111/brv.12315>. *Biological Reviews*. 2017/11/01 2017;92(4):1941-1968. doi:<https://doi.org/10.1111/brv.12315>

33. Stensrud MJ, Hernán MA. Why Test for Proportional Hazards? *JAMA*. 2020;323(14):1401-1402. doi:10.1001/jama.2020.1267
